# A Milk and Ochre Paint Mixture Used 49,000 Years Ago at Sibudu, South Africa

**DOI:** 10.1371/journal.pone.0131273

**Published:** 2015-06-30

**Authors:** Paola Villa, Luca Pollarolo, Ilaria Degano, Leila Birolo, Marco Pasero, Cristian Biagioni, Katerina Douka, Roberto Vinciguerra, Jeannette J. Lucejko, Lyn Wadley

**Affiliations:** 1 University of Colorado Museum, Boulder, Colorado, United States of America; 2 School of Geography, Archaeology and Environmental Studies, University of the Witwatersrand, Johannesburg, South Africa; 3 Istituto Italiano di Paleontologia Umana, Rome, Italy; 4 Laboratoire Archéologie et Peuplement de l’Afrique, Department of Genetics and Evolution, University of Geneva, Geneva, Switzerland; 5 Dipartimento di Chimica, Università di Pisa, Pisa, Italy; 6 Dipartimento di Scienze Chimiche, Complesso Universitario Monte S. Angelo, Napoli, Italy; 7 Dipartimento di Scienze della Terra, Università di Pisa, Pisa, Italy; 8 Research Lab for Archaeology and the History of Art, University of Oxford, Oxford, OX1 3QY, England; 9 Evolutionary Studies Institute, Centre of Excellence, University of the Witwatersrand, Johannesburg, South Africa; Universidade do Algarve, PORTUGAL

## Abstract

Gas chromatography/mass spectrometry, proteomic and scanning electron microscopy with energy-dispersive X-ray spectroscopy (SEM/EDS) analyses of residue on a stone flake from a 49,000 year-old layer of Sibudu (South Africa) indicate a mixture of ochre and casein from milk, likely obtained by killing a lactating wild bovid. Ochre powder production and use are documented in Middle Stone Age South African sites but until now there has been no evidence of the use of milk as a binder. Our analyses show that this ochre-based mixture was neither a hafting adhesive nor a residue left after treating animal skins, but a liquid mixture consisting of a powdered pigment mixed with milk; in other words, a paint medium that could have been applied to a surface or to human skin. The significance of our finds also lies in the fact that it establishes the antiquity of the use of milk as a binder well before the introduction of domestic cattle in South Africa in the first millennium AD.

## Introduction

Paint is a mixture of solid pigment and a liquid vehicle that can be applied to a surface or to a body for decorative or protective [1] purposes. We report here an early case of a paint, preserved as a mineral and organic residue on the working edge of a stone flake from a Middle Stone Age (MSA) layer of Sibudu (South Africa), dated to c. 49,000 years ago [2]. Gas chromatography/mass spectrometry (GC/MS), proteomic and SEM/EDS analyses indicate a mixture of ochre and casein from bovid milk [3]. The presence of bovid milk is surprising at this time since domestic cattle are documented in South Africa only in the Iron Age, about 300 AD [4]. However, hunting practices in the MSA suggest that milk could be obtained by killing a lactating female wild bovid [5]. The novelty of our discovery is highlighted by what is known about ochre use from previous research.

Ochre use is ancient, predating anatomically modern humans in both Europe and Africa. Ochre, possibly in a liquid solution, was used by Neanderthals at Maastricht-Belvédère, Netherlands, 200–250 ka BP [6]. Use of ochre in the African Middle Pleistocene is clearly documented, as shown for example at site GnJh-15 in the Kapthurin Formation, Kenya, 285 ka BP [7], in the 250 ka BP Lupemban Industry of Twin Rivers, Zambia [8], and in the early MSA at Sai Island, Sudan, 200 ka BP [9]. There is an increase in the collection and use of ochre showing grinding, scraping or scoring marks at final Middle Pleistocene sites such as Border Cave layers 5BS and 5WA [10–11] and Pinnacle Point Cave 13B, South Africa (164 ka BP) [12–13]. In the Late Pleistocene use of ochre is a common feature of the South African MSA. Discoveries of ground ochre pieces, ochre powder or traces of ochre on grindstones or stone tools have been made at sites such as Apollo 11 [10] Die Kelders [14], Blombos Cave [15–16], Diepkloof [17], Hoedjiespunt 1 [18], Hollow Rock Shelter [19], Klasies River [20–22], Klipdrift [23], Rose Cottage [24] and Sibudu [25–26]. Ochre pieces are also common in all of the South African Cape west coast MSA shell middens [27]. Deliberately engraved and incised ochre pieces have been found at Blombos and Klasies River Cave 1 [22, 28–29].

Ochre powder is excellent for tanning hides because it reverses the process of decay [30–31] but only circumstantial evidence implies the use of ochre for hide preparation in the MSA. Ochre-stained bone awls from Blombos Cave are thought to have pierced hides, based on experimental replications [16]. Residues and use wear on three Sibudu scrapers tentatively imply their use with ochre on hides [32] but a larger sample size is needed. Ochre had other potential roles in the MSA: ochre nodules seem to have been used as soft stone hammers [33] and at MSA sites like Rose Cottage, Sibudu and Umhlatuzana ochre powder was used in compound adhesives for hafting stone tools [24, 34–35].

Watts [13] suggests that the Pinnacle Point red ochre powders were intended as ingredients for body paints used during ritual performance. The inference of early paint manufacture has some support because an ochre-rich compound that may have been blended with marrow or fat was found stored in two Blombos abalone shells with ages of 101 *±* 4 ka BP [36]. The purpose of such paint remains speculative, but Henshilwood and colleagues propose that possible uses include decoration of various surfaces and skin protection. Some types of ochre are effective sun screens [1]. Ochre traces on the surface or inside perforated marine shells from Blombos (more than 40 *Nassarius kraussianus* shell beads from the Still Bay layers) Sibudu (three perforated *Afrolittorina africana* from the Sill Bay layers) Border Cave (the *Conus* shell associated with the burial of BC3 an infant with an estimated date of c. 76 ka corresponding to the Howiesons Poort at the site) and North African sites suggest that they were worn against painted bodies, but it is also possible that either the shells or strings for threading them had been deliberately covered with red ochre [37–42].

## Materials and Methods

**Fig 1:**
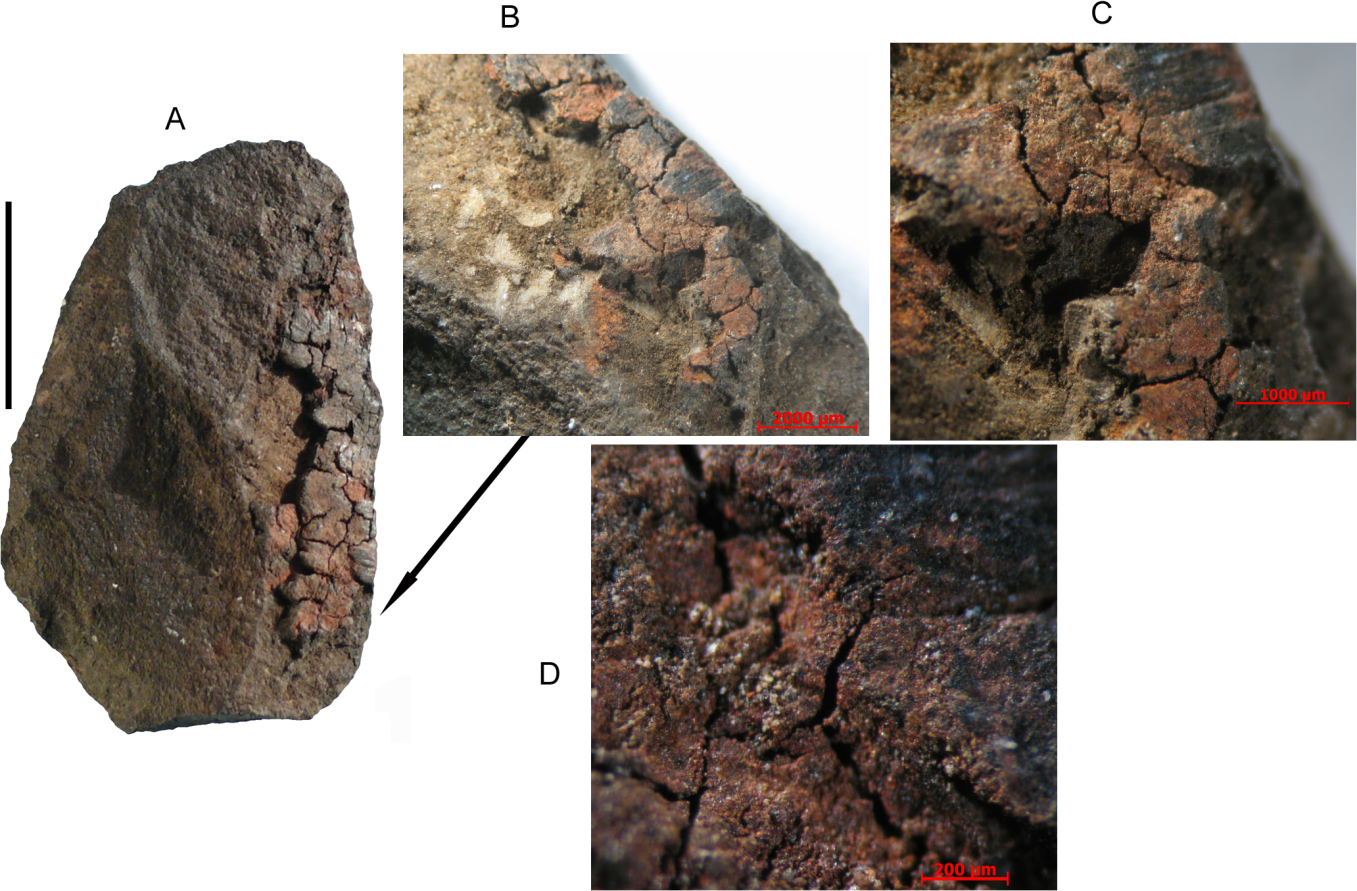
(A) MOD flake before sampling, scale bar = 1 cm. (B) Detail of residue left after sampling for chemical and proteomic analyses. (C) View at 40 x. (D) View at 128 x.

Sibudu is a large rock shelter, approximately 40 km north of Durban and 15 km inland from the Indian Ocean. It has a 2.7 m deep MSA sequence spanning from ca. 77,000 to 38,000 years ago excavated from 1998 to 2011 under the direction of Lyn Wadley [43] (S1 Text. Site setting and excavation). The stone specimen, an unretouched flake of dolerite (length = 26.7 mm; width = 17.5 mm; thickness = 5.1 mm) was recovered from layer MOD dated by single grain OSL to 49.4 ± 2.1 ka [2]. The residue is along the thin, unretouched, right lateral edge (Fig 1A).

Dolerite is an igneous rock that occurs in the vicinity of the site [44]. The flake was found in square D6, quadrant d, where 98 flakes of dolerite were also found. Square D6 yielded 19 pieces of unutilized and 8 pieces of utilized ochre [45] 405 blades and flakes, 3 cores and 32 retouched pieces. In layer MOD there is a total of 192 ochre pieces.

The excavation permit (number 007/09) was issued by Amafa KwaZulu-Natal Heritage Agency in accordance with KwaZulu-Natal Heritage Act 4 of 2008. The permit holder is Lyn Wadley. The Sibudu collections are housed in the Acacia unit of the Evolutionary Studies Institute at the University of the Witwatersrand and are catalogued 2931CA. The export permit of the flake and other pieces for analyses was issued by Amafa KwaZulu-Natal no. 0011/02. Analysis of the MOD lithic assemblage is provided in S2 Text.

### Analysis of the pigment

A small fragment of the residue (dimensions ca. 0.05 x 0.05 x 0.01 mm^3^) was carefully hand-picked and mounted on a Gandolfi camera (R = 57.3 mm) for collecting the powder X-ray diffraction pattern using Ni-filtered Cu *K*α radiation. Owing to the small dimensions of the sample, a 7-day exposure time was set. However, even after 7 days we did not register any sign of diffraction. We tried to capture some weak diffracted spots by means of an air-cooled CCD detector (more sensitive with respect to the photographic film used with the Gandolfi camera) mounted on a Bruker Smart Breeze diffractometer, by rotating the same crystals for several minutes along the φ axis with monochromatized Mo *K*α radiation. However we still did not get any diffraction. This means that the pigment (Fig 1B–1D) consists of non-crystalline or poorly crystalline material.

**Fig 2:**
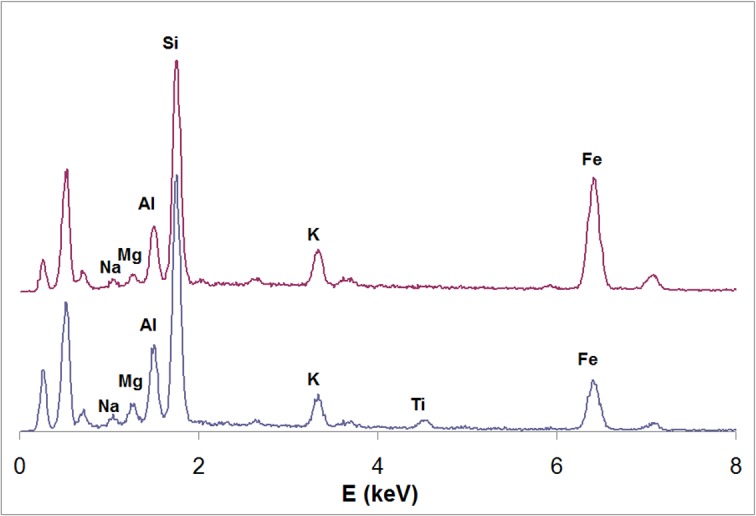
EDS spectra of the residue.

The same fragment was studied with a Philips XL30 Scanning Electron Microscope (SEM) equipped with an EDAX DX4 Spectrometer for chemical (elemental) analysis. A total of 5 spot analyses were performed. All spot analyses showed the same elements (in order of decreasing relative abundance): Si, Fe, Al, K, Mg, Na, and minor Ti. The analyses can be grouped in two subsets, with a different ratio between iron, on one side, and the other major elements, on the other (Fig 2).

We conclude that the pigment consists of a mixture, in variable proportions, of ochre which gives rise to the peak of Fe, and a phyllosilicate possibly belonging to clay minerals, e.g. illite/montmorillonite, giving rise to the other peaks. A shale quarry, likely source of clayey ochre, is 1 km from the site [45].

### Gas chromatography/mass spectrometry

A micro-sample (3.3 mg) of the amorphous residue on the MOD flake was analyzed by gas chromatography/mass spectrometry (GC/MS) using a combined procedure for the identification of lipids, waxes, proteins, resinous materials and polysaccharides possibly present in the same sample (S4 Text, Chemical analyses). To rule out the possibility of contamination due to burial conditions, some milligrams of sediment were subjected to the same combined analytical procedure.

#### Protein fraction (analysis of amino acids)

The amount of amino acids (0.77 μg) in the sample was significantly above the quantitation limit of the procedure (0.33 μg). The amino acidic profile (Table 1 below and Fig A in S4 Text) is consistent with the presence of casein, given the high percentages of leucine, proline and glutammic acid.

**Table 1:** Amino acidic profile of the MOD flake residue.

AA	%
Alanine	6.6
Glycine	8.6
Valine	11.1
Leucine	18.5
Isoleucine	8.4
Serine	6.4
Proline	16.8
Phenylalanine	6.6
Aspartic acid	5.0
Glutammic acid	12.1
Hydroxyproline	0.0

**Fig 3:**
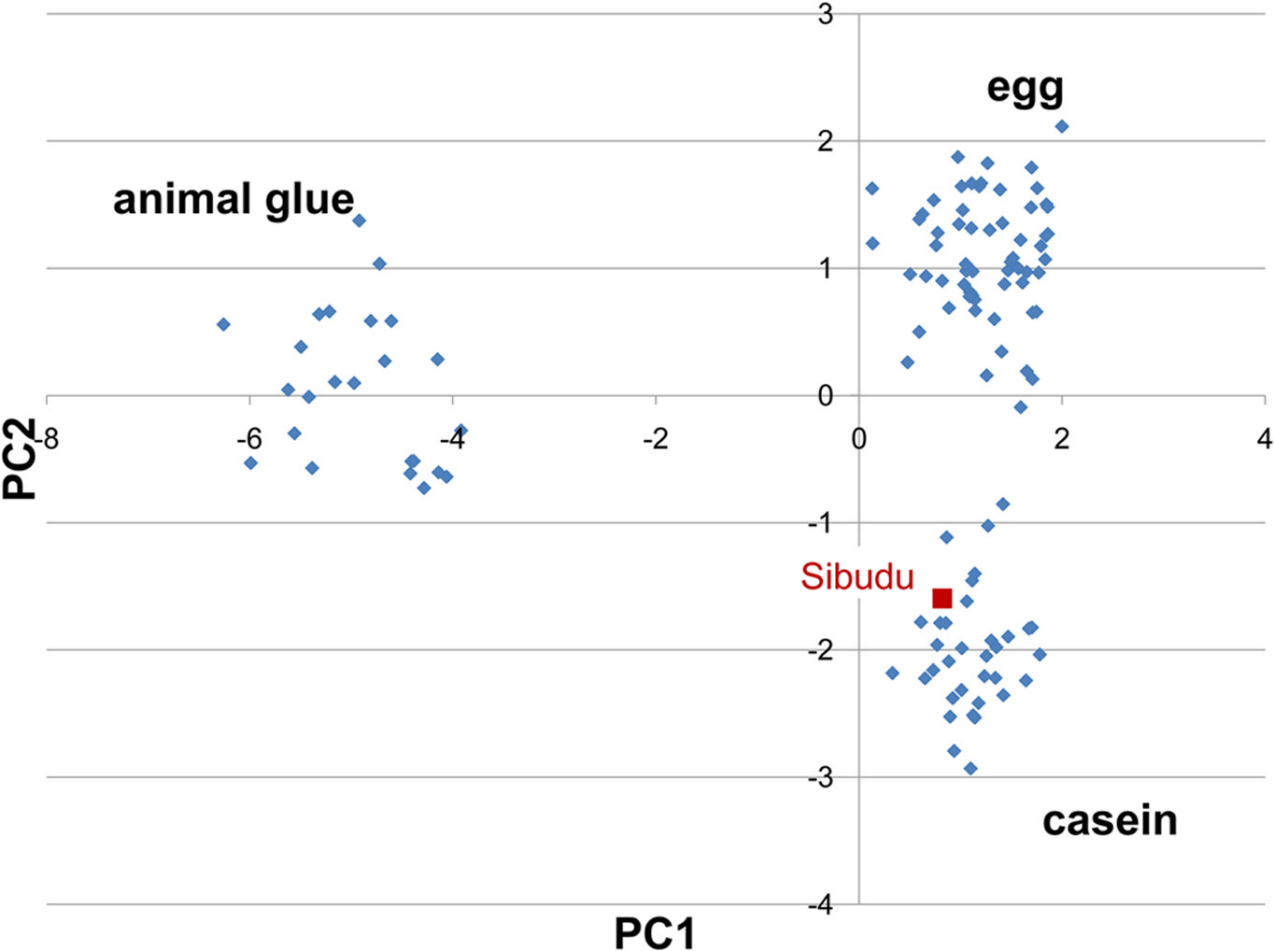
PCA score plot of the database samples and Sibudu sample marked in red.

This was confirmed by submitting the amino acidic profile of the sample to Principal Component Analysis (PCA) together with a dataset of more than 100 reference samples of animal glue, casein, and egg, analyzed as raw materials and paint layers mixed with pigments, both naturally and artificially aged. The resulting score plot is reported in Fig 3, and shows that the sample is located in the cluster of casein. The absence of animal glue is confirmed by the absence of hydroxyproline. The amount of amino acids in the soil sample was below detection limit.

#### Polysaccharide fraction (analysis of sugar and uronic acids)

The analysis of the sugar and uronic acids fraction did not yield significant results: the amount of analytes was below the quantitation limit of the procedure (0.6 μg).

#### Lipid and resinous fraction

Several fatty acids were identified (Table A and Fig B in S4 Text). In particular, linear monocarboxylic acids C12-C24 with a minor amount of azelaic acid and glycerol were detected, suggesting the presence of a lipid of plant origin. Sediment samples were used as analytical blanks. The amount of fatty acids in the sediment samples was several orders of magnitude lower than that in the flake sample. Therefore, we can exclude soil contamination. However, the flake fatty acid profile may have been altered by bacteria present in the sediment. Thus, we cannot be confident that the lipid material was actually of plant origin.

In conclusion, the occurrence of casein was inferred from the PCA score plot. This interpretation was tested and confirmed by proteomic analyses.

### Proteomic analyses

Proteins in the sample were identified following a minimally invasive proteomic procedure using a 4 mg sample of the residue (S4 Text, Chemical analyses). Raw data from nano liquid chromatography-tandem mass spectrometry (LC-MS/MS) were used to query the Swiss Prot database which contains protein sequences of modern species. A database search provided a good score for four bovine milk caseins: Alpha-S1-casein, (P02662), Alpha-S2-casein, (P02663), Beta-casein, (P02666), and Kappa-casein, (P02668). Two collagens [Collagen alpha-1(I) chain, (P02453), and Collagen alpha-2(I) chain, (P02465)] found in bovine species were identified in the sample (Table C in S4 Text).

Proteomics identification is straightforward but it relies on the presence of the sequence to be matched in the database or, at least, that a highly homologous protein from another species is available. Some peptides identical to the ones present in the known protein homologue are then expected to be produced in the enzymatic digestion of the protein of interest and can be matched. Although casein proteins are highly divergent across mammalian milks [46], they are conserved enough to allow identification, with some peptides identical to the ones present in the old protein homologue. Divergence, moreover, allowed genus-specific sequence information, since several of the matched peptides are specific enough to allow us to confidently identify the bovid origin of the caseins. In Fig F of S4 Text the alpha-S1 casein sequences from *Bos taurus* were aligned to those from some other potential sources of milk, i.e. human, sheep and goat. The matched peptides in the MOD flake (highlighted in grey and bold) clearly indicate that the casein belongs to an animal in the bovid family. Likewise, alignments of the other identified caseins with homologous proteins are similarly reported by [47] showing that several of the identified peptides are proteotipic of bovine caseins sequences.

Collagen was not identified in the fraction of the sample that was analyzed by GC/MS given the absence in the amino-acidic profile of hydroxyproline which is a marker of animal glue. This indicates that collagen was not uniformly mixed in the sample as one would expect if collagen was used as animal glue and that the collagen source was most likely a minute bone fragment or a fragment of connective or epithelial tissue (see Discussion).

Type I collagen is sufficiently variable between mammal genera to be taxonomically useful. Collagen from several bone samples of equid and bovid fauna from layer MOD was extracted and analyzed. Peptide matches from each sample and from the MOD flake were manually examined and aligned to the collagen sequences from modern species. Significantly comparisons with samples of equid and bovid fauna from layer MOD indicate that the collagens were from a bovid and not from an equid (Figs G and H in S4 Text).

In sum, independent analyses by two laboratories on different fraction of the residue and with different techniques support the identification of casein. Moreover proteomic analysis shows that the casein belongs to an animal in the bovid family.

### Direct dating

Following the GC/MS and proteomic analyses which suggested the presence of organic material still preserved in the residue, the sample was considered suitable for direct AMS dating. The analysis was performed at the Oxford Radiocarbon Accelerator Unit (ORAU), University of Oxford, UK.

The direct AMS dating of the residue is 23.3 ± 0.4 ka BP (uncalibrated radiocarbon years before present). Lack of chemical cleaning, a decision imposed by the very small size of the sample (13 mg), meant that any contaminants would remain intact contributing to the overall ^14^C measurement. The determination therefore is considered minimum age only. The larger than normal standard error is a factor of the small amount of C measured in the sample (390μg), about 5 times smaller than the normal-sized graphites routinely measured at the ORAU.

The significance of this direct measurement lies in the fact that it establishes the antiquity of the use of milk well before the introduction of domestic cattle in South Africa in the 1st millennium AD (S3 Text. Direct dating).

## Discussion

Milk is an emulsion of fats within a water-based fluid which also contains carbohydrates, proteins and minerals. Casein is the major protein component of milk and is extracted by heating milk and adding acid [3]. Casein then precipitates into small, solid globules. Use of casein as a glue requires mixing with quicklime or hydrated lime (calcium hydroxide, also called slaked lime) [48]. Lime is obtained by crushing and heating limestone. However, recent geological mapping shows that there is no limestone in the Sibudu area [49]. For use as a binder for pigments, lumps of casein can also be dissolved and thinned with water and wood ashes (mainly composed of calcium carbonate or calcium oxide). However calcium (Ca) is absent in the spectra of Fig 2. Given the absence of lime and wood ashes in the Sibudu residue, we reject an interpretation of the casein as either glue or a complex form of binder. Instead, we conclude that powdered ochre was simply mixed with milk in its liquid form and casein is the residue left after evaporation, since milk is a water-based liquid.

The identification of a milk and ochre mixture on a 49,000 year old flake from a late MSA layer of Sibudu raises a number of questions indicated below.

### How was the milk obtained from wild bovids?

Some African bovids separate from the herd when giving birth and hide their calves until they are strong enough to keep up with the herd as a protection against carnivores [50]. This is the case with the eland (*Taurotragus oryx*), the kudu (*Tragelaphus strepsiceros*), and the impala (*Aepicerus melampus*). The bushbuck (*Tragelaphus scriptus*) and the red and blue duiker (*Cephalophus natalensis* and *Philantomba monticola*) are solitary animals; they hide their young and go off to browse alone. All these species and the buffalo (*Syncerus caffer*, a herd animal) are represented in the MOD fauna.

Klein [5] notes that in the MSA deposits of Klasies River Cave I the giant buffalo (*Pelorovis antiquus*) a very large bovid with a horn span of 3 m or more, falls into two age groups: newborn or perhaps fetal individuals and physically mature individuals with worn molars. According to Klein, the explanation for this peculiar distribution may be that the Klasies people, faced with such a formidable prey, focused on females in advanced pregnancy or in the process of giving birth. Cows are easy prey when giving birth and would already have milk. It would not be difficult for hunters to locate lactating cows, particularly among seasonal breeders. This hunting pattern would result in access to milk and might explain the inhomogeneous presence of collagen observed by proteomic analyses. In the process of collecting milk from the mammary glands of a dead animal, a small amount of its epithelial tissue could have been taken as well.

### Was the mixture a hafting adhesive?

The use of adhesives made from resin or plant gum combined with ochre has previously been suggested for the hafting of stone points and backed pieces to be used as hunting tools at Sibudu. Howiesons Poort and later MSA tools were studied by optical microscopy in combination with spatial distribution of residues on the tool [35, 51]. Replication experiments [34, 52] demonstrated the usefulness of ochre as a loading agent with products such as plant gum. Microscopic analysis showed that ground ochre was mixed with a plant exudate; experiments suggested that ochre-loaded adhesives are robust, dry fast and are not hygroscopic. Most archaeological residues occurred at the base of the point or on the back of backed pieces implying that the mixture was used for hafting. However optical microscopy alone cannot securely distinguish between plant gum (polysaccharides) and resin (terpenoid secretions from trees) [53] thus new analyses using GC/MS are appropriate.

The residue on the MOD flake occurs on the thin edge of an unretouched flake, not on the back or proximal part of the piece as would be expected if used for hafting. An alternative explanation is that the flake was used to prepare or apply the ochre-loaded mixture. GC/MS has been used to analyze six tools, dated between 65 and 38 ka BP, from Sibudu (n = 5) and Rose Cottage (n = 1). The residues were preliminarily ascertained by visual inspection. All samples were provided by Lyn Wadley. The purpose was to identify the residues on the MSA tools and to determine whether casein could have been used as glue in a variant of the recipes used at Sibudu.

The results (S4 Text, Chemical analyses) indicate that two of the Howiesons Poort segments, from layer GR in square C6c and from layer PGS in square B5a, contained a relevant amount of diterpenes indicating the use of a conifer resin, possibly from *Afrocarpus* (syn. *Podocarpus*) *falcatus*, and some lipid material. There is neither evidence of plant gums (no polysaccharides) nor proteinaceous material such as casein. No significant molecular markers of organic material were detected on the other samples; neither saccharides nor terpenes were detected. Fatty acids were present in very low quantities suggesting soil contamination. The use of conifer resin for hafting (*Podocarpus elongatus*) is also indicated by GC/MS analysis on a quartz backed flake dated to ca. 56 ± 10 ka BP in the Late Howiesons Poort at the site of Diepkloof [54].

As mentioned before, the use of casein as a glue requires mixing with hydrated lime [48] which is obtained by crushing and heating limestone. Recent geological mapping shows that there is no limestone in the Sibudu area [49] so, in conclusion, there is no evidence that the MOD flake residue was a hafting adhesive.

### Was the MOD flake used to treat animal skin?

Three scrapers from layer SS at Sibudu, dated to 59.6 ± 2.3 ka by OSL [2], have ochre and animal products (fat, muscle tissue) on their edges. Ochre and fat were quite probably used for processing hides and some of the animal products were the result of use on animal skin [32]. The presence of milk is however not consistent with use for processing hides.

### What was the use of the MOD flake mixture?

Our analyses show that this ochre-based mixture was neither a hafting adhesive nor a residue left after treating animal skins, but a liquid mixture consisting of a solid (powdered) pigment mixed with milk; in other words, a paint medium that could have been applied to a surface or on the skin.

The edge of the flake may have been used as a mixing implement for combining the substances. A mixture of milk and ochre may have been used for body painting, skin protection or for painting on a rock surface like a stone slab. The oldest representational rock art in Africa is on seven small quartzite slabs from the Apollo 11 rock shelter in Namibia. The date of 27,500 years ago is an average of 3 radiocarbon samples of charcoal in hearths from the same layer [55]. The pigments and binders of the slabs have not yet been analyzed. Traces of red coloring on ostrich eggshell fragments from the older MSA layers in Apollo 11 [56] imply that there may have been simpler precursors to the representational art. Milk or casein has never been documented as media for pigment in San rock art [57]. In fact, little is known about the methods or ingredients that San used for making paint and even less is known about paint binders [58–61]. Blood was identified in rock art paint at Rose Cottage [62]. Some ethnographies and historic sources mention milk and egg as a binder for rock paint but there is as yet no direct evidence for its use [57].

Milk was not used as a binder with pigments for body paint by the San (Bushman) nor by the Hottentots and Nama herders of South Africa, Namibia and Botswana; animal or vegetable fat were the most commonly reported media for body paint [58]. However clarified butter is mixed with ochre for body paint used by the Himba of Namibia [1]. Only further research on pigments and binders of rock art in South Africa will allow us to identify similarities or differences that may support one hypothesis over the other.

Nevertheless, obtaining milk by killing a lactating wild bovid and then mixing it with ochre shows that MSA people experimented with coloring materials in creative ways and may have attributed a special significance and value to that product. In the present state of research we cannot demonstrate conclusively whether paint such as that found at Sibudu was a precursor to rock art painting or used for body painting. In either case this find suggests that toward the end of the Middle Stone Age new techniques of symbolic communication were evolving that became, some millennia later, key elements in the social life of hunter-gatherers in South Africa.

## Supporting Information

S1 Text Site setting and excavation (PDF)

S2 Text Lithic analysis of layer MOD (PDF)

S3 Text Direct dating (PDF)

S4 Text Chemical analyses (PDF)
