# Supplementary material for: A Milk and Ochre Paint Mixture Used 49,000 Years Ago at Sibudu, South Africa
Source: PLoS One. 2015 Jun 30;10(6):e0131273. doi: 10.1371/journal.pone.0131273 (PMC4488428; doi:10.1371/journal.pone.0131273)
Supplement: S1 Text — (PDF) [file pone.0131273.s001.pdf]

Supporting Information

A milk and ochre paint mixture used 49,000 years ago at Sibudu, South Africa

Paola Villa, Luca Pollarolo, Ilaria Degano, Leila Birolo, Marco Pasero, Cristian Biagioni, Katerina Douka, Roberto Vinciguerra, Jeannette J. Lucejko, Lyn Wadley

S1 Text. Site setting and excavation

Sibudu is a large rock shelter situated in Kwa-Zulu Natal, on a cliff above the Tongati (also spelled as u-Thongathi) river, in the sandstone and shales of the Natal Group (Figs.A-B). It has a long and detailed Middle Stone Age (MSA) sequence (Fig. C) with excellent organic preservation including plant materials [1].

Layer MOD, a mottled brown silty sand with flecks of white ash, gypsum and charcoal, about 10-15 cm thick, was excavated on a surface of 11 square meters. It is dated by OSL to 49.1 ± 2.1; the weighted mean OSL age for layers RSp, OMOD, BMOD and MOD in squares B5-B6 is estimated at 48.0 ± 1.4 [2-3] (Table A). The excavation squares, each 1 sq m, are subdivided in four 50 x 50 cm quadrants named a, b, c, d.

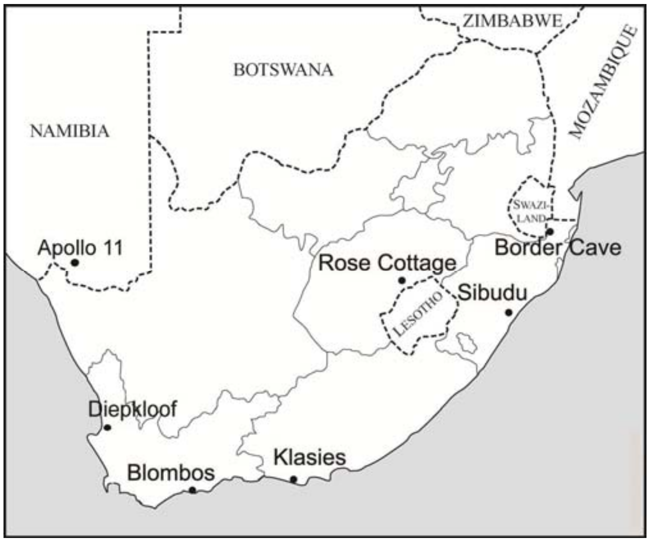

Fig. A. Map with location of Southern African sites mentioned in the text.

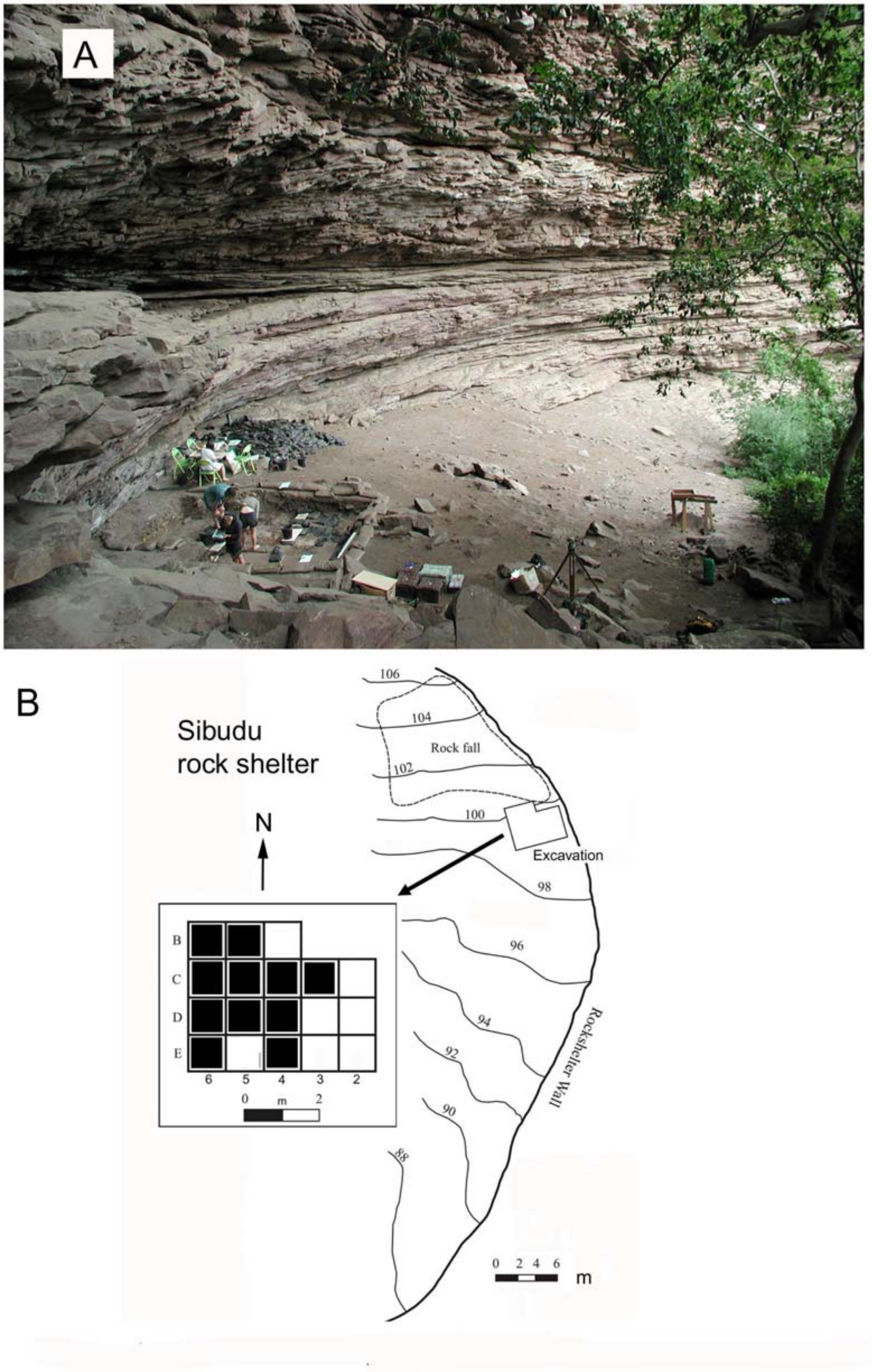

Fig. B. (A) Sibudu rock shelter during excavation. (B) Plan of the site and squares excavated in MOD. The site grid is in 1-meter squares.

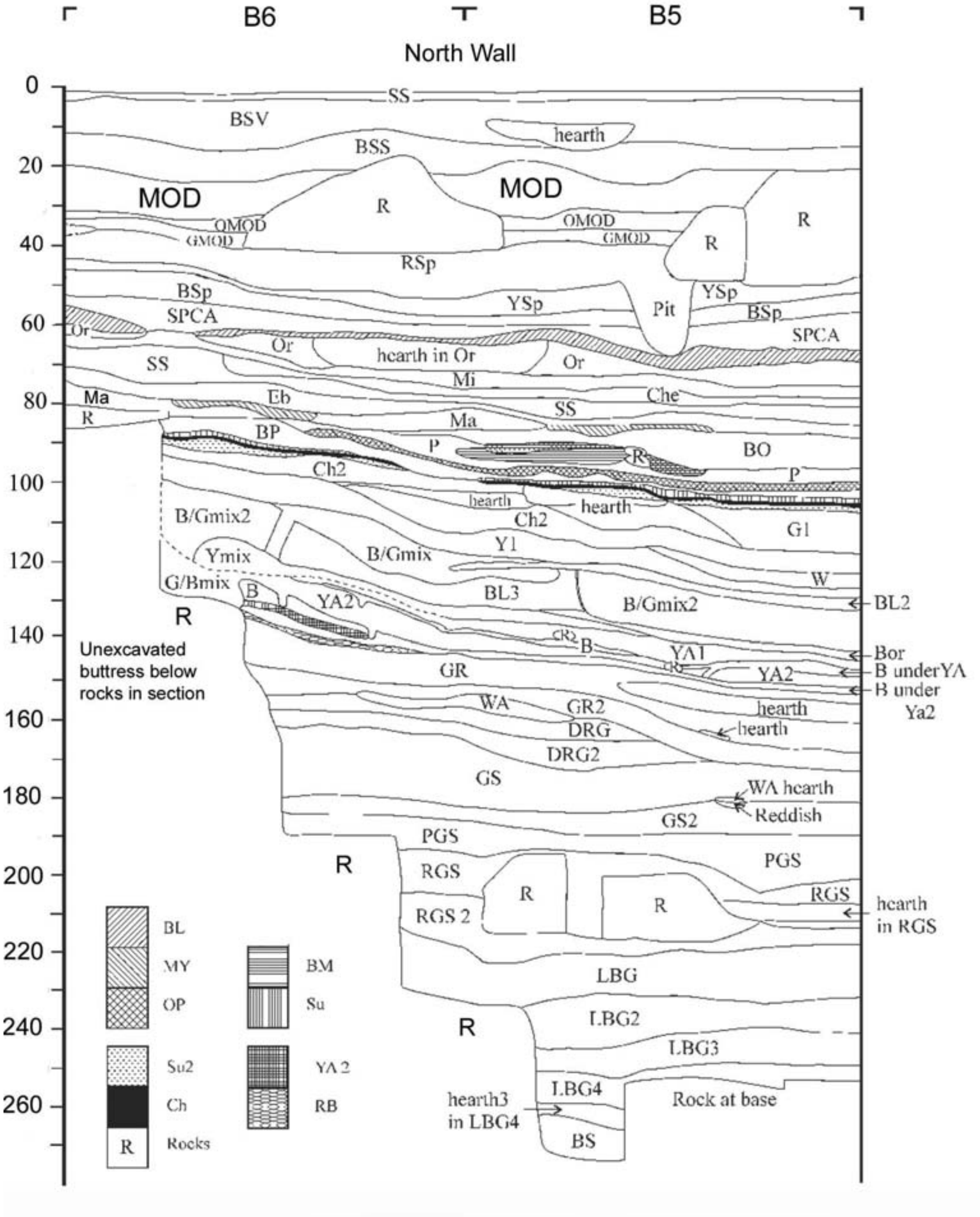

Fig. C. Sibudu stratigraphic section on the north wall. Layer MOD is at the top of the Middle Stone Age sequence and below the Iron Age layers BSS and BSV. Modified after [4].

Table A. Late and Final MSA layers at Sibudu [2]. They are in stratigraphic sequence together with their OSL ages. Layers Ore to Co are at the very top of the sequence and are not represented in the North section. Layer MOD is present only in the North section.

East Section	OSL age (ka)	North Section	OSL age (ka)
Co	38.0 ± 2.6		
Bu	39.1 ± 2.5		
LBMOD	49.9 ± 2.5		
MC, Es			
Mou, DMou, LMou			
Ore, PB			
		MOD	49.1 ± 2.1
		OMOD	46.6 ± 2.3
		BMOD	
		RSp	48.0 ± 1.4 ka, weighted mean

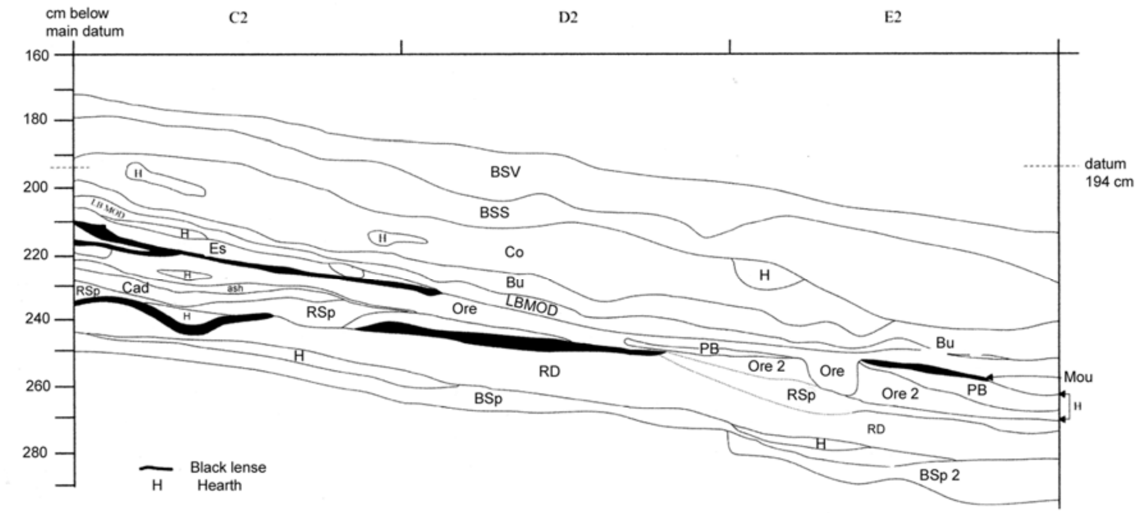

Fig. D. Sibudu, stratigraphic section of the eastern wall. The black strata are hearth lenses. Modified after [5].

References

1. Wadley L, Sievers C, Bamford M, Goldberg P, Berna F, Miller C. Middle Stone Age bedding construction and settlement patterns at Sibudu, South Africa. Science. 2011; 334: 1388-1391.

2. Jacobs Z, Wintle AG, Duller GAT, Roberts RG, Wadley L.New ages for the post-Howiesons Poort, late and final Middle Stone Age at Sibudu, South Africa. J Archaeol Sci. 2008; 35: 1790-1807.
3. Wadley L, Jacobs Z. Sibudu Cave: background to the excavations, stratigraphy and dating. South Afr Humanit. 2006; 18: 1-26.
4. Wadley L. The Howieson's Poort industry of Sibudu Cave. South African Archaeological Society, Goodwin Series 2008; 10: 122-132.
5. Wadley L. A typological study of the final Middle Stone Age stone tools from Sibudu Cave, KwaZulu-Natal. S Afr Archaeol Bull. 2005; 60 : 1–13.
