# Supplementary material for: A Milk and Ochre Paint Mixture Used 49,000 Years Ago at Sibudu, South Africa
Source: PLoS One. 2015 Jun 30;10(6):e0131273. doi: 10.1371/journal.pone.0131273 (PMC4488428; doi:10.1371/journal.pone.0131273)
Supplement: S2 Text — (PDF) [file pone.0131273.s002.pdf]

Supporting Information

A milk and ochre paint mixture used 49,000 years ago at Sibudu, South Africa

Paola Villa, Luca Pollarolo, Ilaria Degano, Leila Birolo, Marco Pasero, Cristian Biagioni, Katerina Douka, Roberto Vinciguerra, Jeannette J. Lucejko, Lyn Wadley

S2 Text. Lithic analysis of layer MOD

Sorting and sampling

The MOD layer lithic materials are very abundant and needed to be sorted prior to analysis. Our analyses required a large amount of effort for sorting and bagging in transparent ziplock bags with preprinted labels prior to technological analysis and coding in Excel files. The flake with residue was discovered during sorting by one of us (LP).

To facilitate inter-assemblage comparisons we use the same sorting procedures also used at other South African sites (Rose Cottage, Klasies River Cave 1A, Blombos, Border Cave [1-5]). We select all cores, core fragments, core fragments, tools, tool fragments and all blade and blade fragments regardless of size. Retouched pieces and cores are assigned individual catalogue numbers. Complete or broken flakes preserving the platform > 1 cm are also selected. This sorting procedures excludes flake fragments (broken flakes without the platform), flakes < 1 cm and chunks from technological analysis; however the small debris is bagged by large categories and remains available for specific studies. This selection procedure has several advantages: a) it smoothes differences in sorting precision and screen size between excavations, b) it greatly accelerates the analysis of flaking methods by sorting out the less informative small debris and flake fragments, and c) it sets an explicit cut-off point for calculating assemblage composition.

Raw material

Hornfels and dolerite are the two most common raw materials used for the production of blades and flakes and they come from a variety of sources. Dolerite, an igneous rock, is most often coarse-grained but there is also a fine-grained variety. It occurs as dykes and sills in the vicinity of Sibudu The outer surface of the pieces show either fresh cortex or a natural surface (unweathered or only slightly weathered surface corresponding to fissure planes present in the rock) implying that the slab was collected near or at the outcrop. However, alluvial cortex is most common; the rock was probably collected on the banks of the Tongati River, below the site. In layer MOD dolerite represents 60% of the debitage (i.e. unretouched flakes and blades, cores and core fragments 611/1030) but only 15% of the retouched pieces (23/154) which were most often made of hornfels.

Hornfels (a fine-grained metamorphic rock) occurs as thin slabs and as rolled pebbles; the closest known outcrop of good-quality hornfels at Verulam on the Black Mhlasini river, about 15 km to the south of Sibudu [6]. Alluvial cortex occurs on the less common quartz and quartzite indicating that some of the sources were rounded nodules or pebbles from river gravels or exposed conglomerates.

Table A. Assemblage composition by raw material

**Category**	**Hornfels**	**Dolerite**	**Quartzite**	**Quartz**	**Other**	**TOTALS**
Blades and blade fragments	128	213	16	1	3	361
Flakes and proximal flake fragments[1]	220	395	20	3	13	651
Bipolar flakes	6	0	0	0	0	6
Cores and core fragments	11	6	0	0	2	19
Bipolar cores	1	0	0	1	0	2
Retouched pieces and fragments	119	23	3	5	4	154
**TOTALS**	485	637	39	10	22	**1194**

[1]We include in the flakes a small number of technical pieces of hornfels (n = 6) and dolerite (n = 3). These are flakes from resharpening of a retouched edge on a unifacial or bifacial tool (recognizable by their platform) and characteristic overshot flakes, knapping accidents which occur during shaping or resharpening of points [7]. “Other” corresponds to sandstone and chert.

Table B. Core types

**Core type**	**Hornfels**	**Dolerite**	**Quartz**	**Chert**	**Sandstone**
Blade core	1	1	0	0	0
Bladelet and blade/bladelet core	4	0	0	1	0
Flake core	3	4	0	0	0
Bipolar core	1	0	1	0	0
Core tablets, indeterminate and fragments	2	2	0	0	1
**TOTALS**	11	7	1	1	1

Both flake and blades reduction sequences are represented (Figs. A-B). Cores with flake negatives only are Levallois recurrent (n =3), cores with a discoid morphology (n = 2) and others less organized. The reduction sequence of blades and bladelets is based on bidirectional flaking from two opposing platforms on the wide core surface and also on the narrow side of the core.

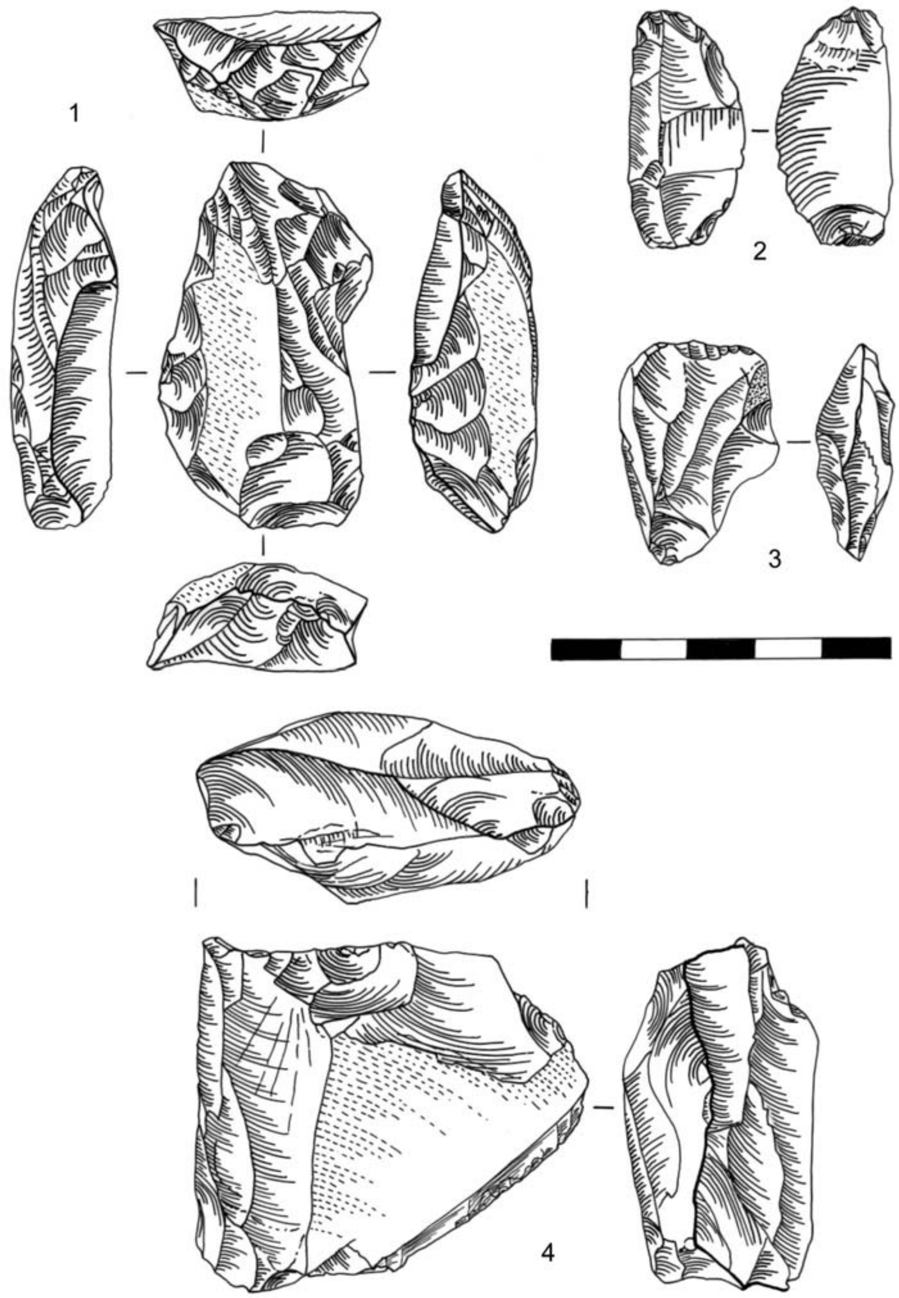

Fig. A. Sibudu layer MOD, cores. (1) Blade/ bladelet core, square B5d, on a hornfels flake from a slab, note the natural surface. Two small blade removals from opposite platforms have width of 9.6 and 8.4 mm, with hinge terminations. (2) Bipolar core on flake, square D4a, hornfels. (3) Bidirectional bladelet core, square D6a, hornfels. The last bladelet is 22 x 5.3 mm; the last removal has a step termination. (4) Bidirectional blade core, abandoned due to many failed step terminations; square D6d, on a hornfels slab. Scale bar in cm.

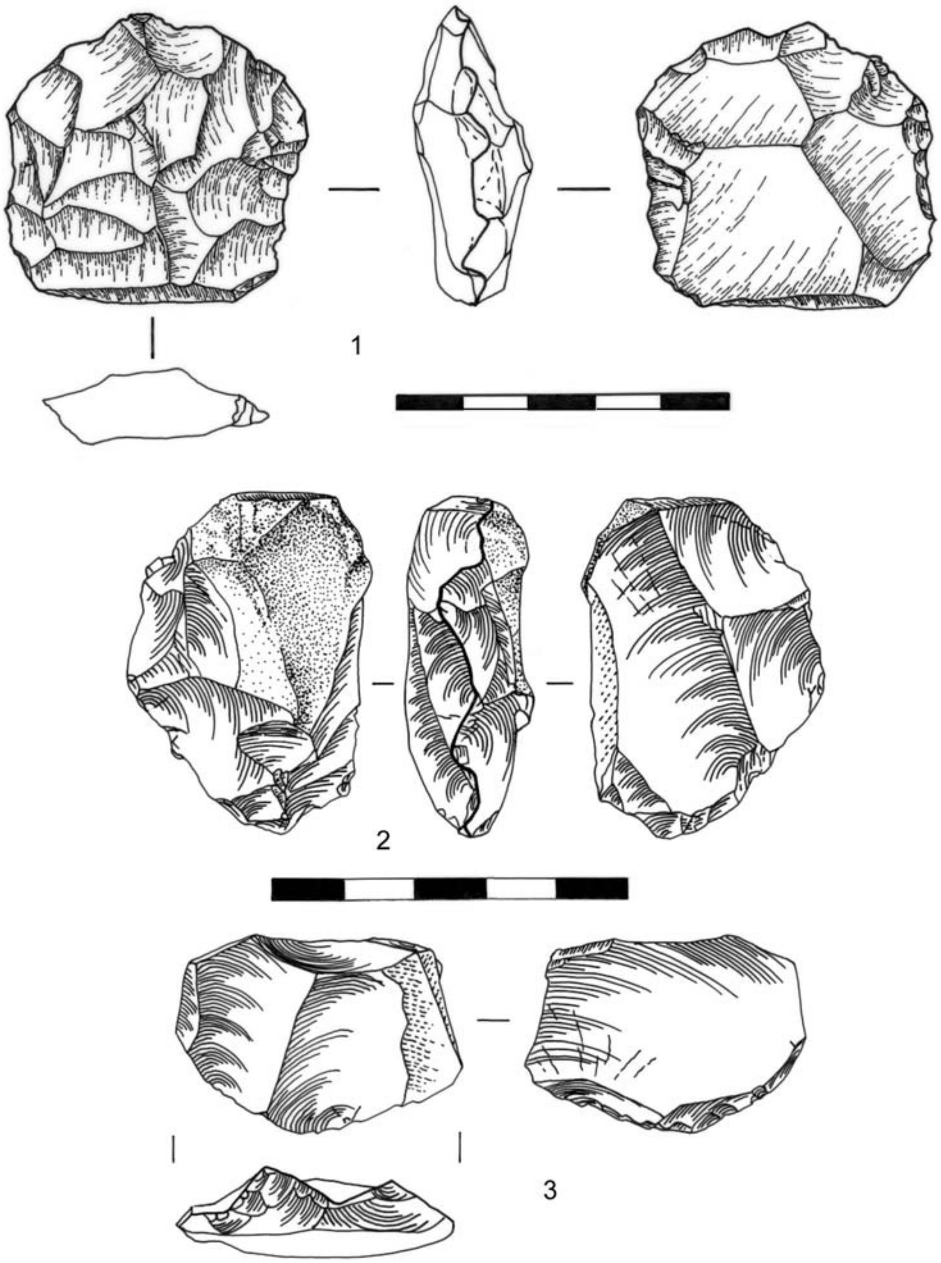

Fig. B. Sibudu layer MOD, cores. (1) Levallois recurrent centripetal core, square E4c, dolerite (2) Core of discoid morphology, square B5c, hornfels. (3) Core tablet from a unidirectional core, square D5b, hornfels. Scale bar in cm.

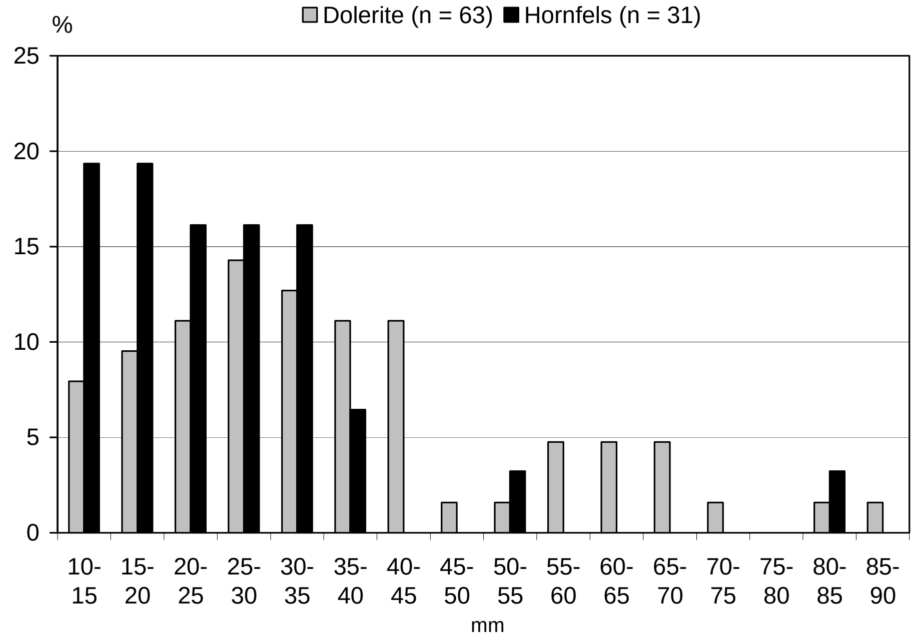

Fig. C. Length of dolerite and hornfels blades**.** Bladelets form a continuum with blades and were rarely selected as blanks for formal tools (there are only two retouched bladelets in the assemblage).

Retouched pieces (Tables C-E)

The production of flakes is predominant in this assemblage. Flakes are the preferred blank and were used for unifacial and bifacial points, scrapers and informally retouched pieces. Blades were sometimes used as blanks for side scrapers and for truncations but were most often only informally retouched. Two retouched bladelets (L = 2 cm) were made of quartz.

Unifacial points are characteristic of this period and are the most common form in this assemblage. They are always made on flakes, three are on convergent elongated flakes with minimal retouch that fit the definition of Levallois (Fig. D). Note, however, that there is no systematic production of Levallois flakes: in the debitage there are only five pseudo-Levallois points and one thick Levallois flake. The few bifacial or partly bifacial points have a broad base. There are no concave-based (also called hollow-based) bifacial points that are markers of the final MSA at Sibudu.

The mean length and standard deviation of unifacial and bifacial points is 47.6 ± 10.3 (n = 20). The value of formulas such as tip cross-sectional area (TCSA) and Tip Cross-Sectional Perimeter (TCSP) for distinguishing between armatures of different weapon systems (whether arrows, spearthrower darts or throwing and thrusting spears [8-9] has been recently challenged by a study of ethnographic Australian stone points hafted as spearthrower darts and as knives [10]. These ethnographic projectile points have significantly higher TCSA values than the expected. The implication is clear: the range and mean values of those formulas will not distinguish spears from darts or even axially hafted knives. However the presence of impact scars on some of the Sibudu MOD points (Fig. D) and their relatively large size strongly suggest that some at least were used as spear points and not as arrowheads. Our data indicates similar

conclusions can be drawn from late MSA layers below MOD (RSp, OMOD) and above MOD (layers in the East Section (Ore to Co; Table A in S1 Text file) [11-12]).

Table C. Frequencies of retouched pieces

**Types of tools**	N	%
Unifacial points	26	16.9
Bifacial and partly bifacial points	11	7.1
End scrapers	4	2.6
Side scrapers	18	11.7
Convergent scrapers	7	4.5
Notches	7	4.5
Denticulates	3	1.9
Retouched blades and bladelets	10	6.5
Retouched flakes	17	11.0
Truncations	3	1.9
Burin	1	0.6
Backed pieces	2	1.3
Bifacial pieces	2	1.3
Piece esquillées	1	0.6
Tool fragments	42	27.3
**Total**	**154**	**100**

Table D. Raw materials of retouched pieces

**Raw Material**	**N**	**%**
Hornfels	119	77.3
Dolerite	23	14.9
Quartz	5	3.2
Quartzite	3	1.9
Chert	2	1.3
Sandstone	2	1.3
**Total**	**154**	**100**

While hornfels was used for all kinds of tools, dolerite was used for unifacial points and informally retouched flakes and blades. Quartzite was used for three unifacial points.

Table E. Blanks of retouched pieces

Type of blank	N	%
Blade	20	18.5
Flake	86	79.6
Bipolar flake	1	0.9
Chunk	1	0.9
**Total**	**108**	**100.0**

Another notable tool form in this assemblage is the backed piece. The term should not be taken to imply any form of continuity between the backed pieces of the Howiesons Poort (HP) layers and the late MSA layers. There are only two backed pieces in layer MOD and they are a form that also occurs in the younger, final MSA layers of the East Section (Table A, Fig. D in S1 Text file); two other pieces are truncations. Three of these pieces are on flakes. The HP backed pieces are predominantly made on blades (96 to 100%) and similar frequencies can be observed at Rose Cottage (96.0%) and Klasies River Main Cave 1A (90.6%). The HP backed pieces are by far the most common tools type and they are elongated forms (Fig. E).
After the HP backed pieces practically disappear. Will et al. [13] counted 4 backed pieces in the post-HP layers prior to layer RSp. There is only one segment in layer RSp which is dated to 48.0 ± 1.4 ka, weighted mean OSL age [14]. Afterwards “backed “pieces are made on flakes and have quite a different morphology (Fig. F). They are rare forms and their function is unknown. Based on the backing and their shape they were perhaps hafted transversely. They are, however, much bigger than Mesolithic trapezes, generally interpreted as transverse arrowheads.

Conclusions
The technology and typology of layer MOD lithics shows similarities but also some differences with other post-HP assemblages such as Klasies River Main Cave 1A, Rose Cottage and Border Cave layer 2WA. As at those sites, backed pieces disappear and triangular points reappear, marking a real discontinuity with the HP. As at Rose Cottage flake production (with a limited use of the Levallois method) is clearly predominant and the frequency of side scrapers rises. However the MOD assemblage shows no evidence for a gradual abandonment of the technology and tool types of the post-HP period and no marked rise in the use of the bipolar knapping, even in the final MSA layers, although these trends are easily observed at Border Cave in layers dated to 45-49 ka [5]. Whether these differences are due to regionally variable trajectories or to imperfect chronologies remains a question for future studies.

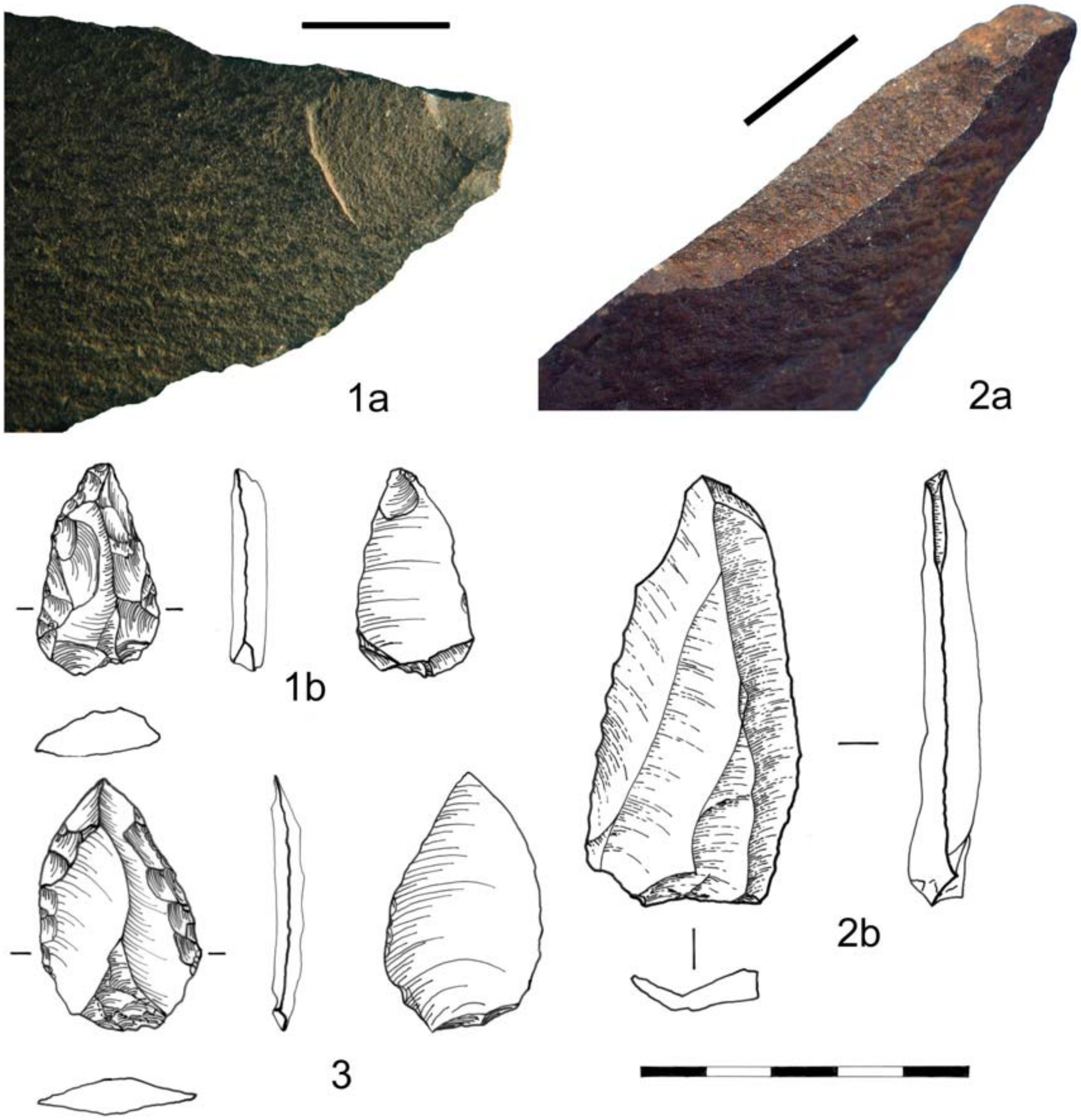

Fig. D. Sibudu layer MOD, unifacial points. (1a,b) Hearth above MOD C4 a+b no. 40 hornfels. The step scar at the tip is 7 mm long and can be interpreted as an impact scar. The scar and the basal thinning indicate axial hafting. (2a,b) MOD, Levallois triangular flake with minimal retouch and a burin-like impact scar at the tip, 17 mm long; square D6d, no. 5, dolerite. (3) MOD square D4d, no. 39, hornfels. Scale bar of photos = 1 cm.

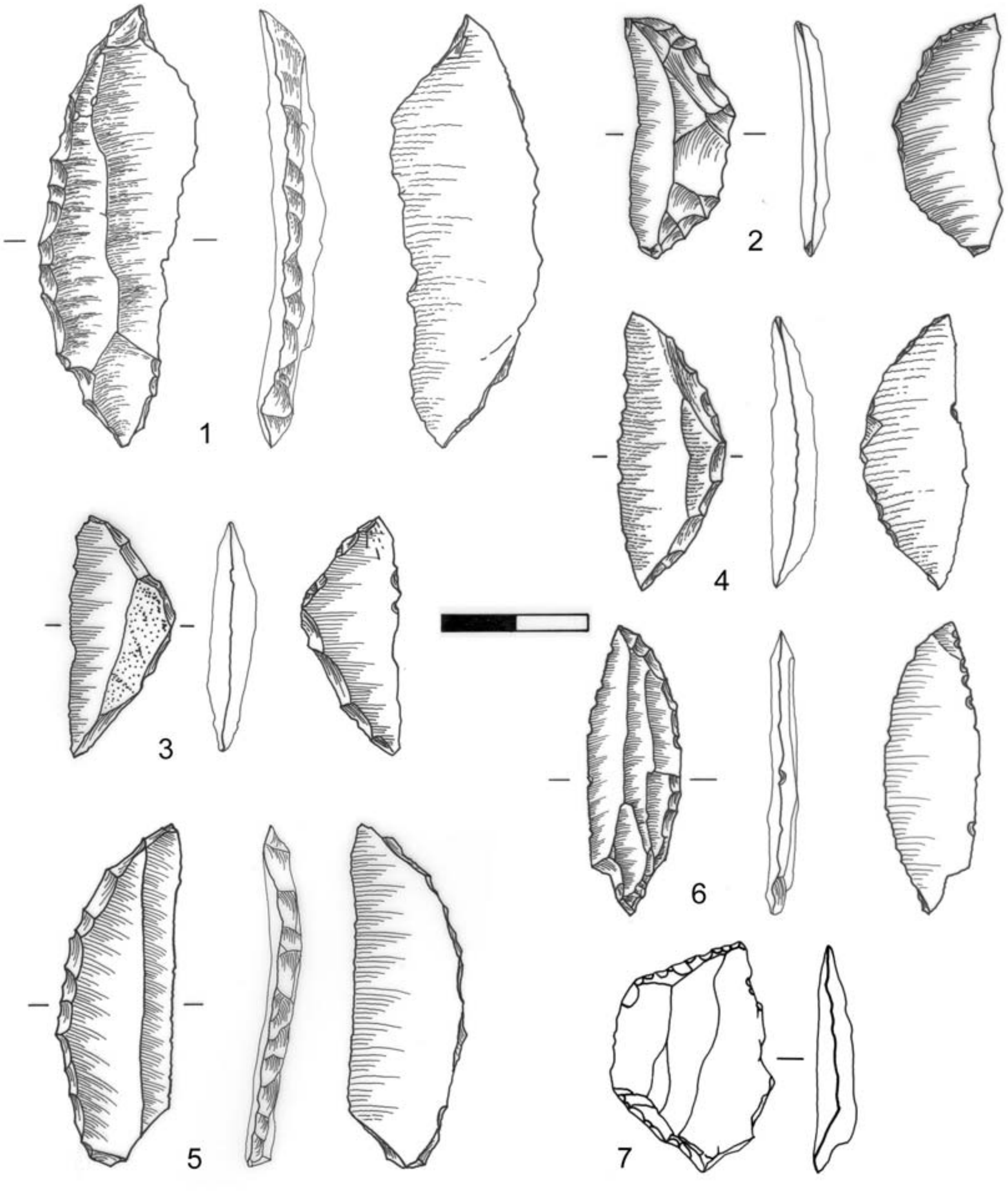

Fig. E. Sibudu, backed pieces in the Howiesons Poort (HP) layers (1-5) and in late MSA layer RSp and BMOD (6-7). The HP and RSp segments are on blades, the BMOD piece is on flake. Pieces are indicated with layer, square and catalogue number. (1) Brown under Yellow Ash 2 (= layer GR) B6a PM3, dolerite. (2) GR B5c M3, hornfels. This is a partly backed piece. (3) GR B5b M5, hornfels. (4) White Ash below GR II (= layer GS) B6a PM12, dolerite. (5) GS B6a PM14, hornfels. (6) RSp C6a, no number, hornfels. The notch at the base is an unusual feature, never observed on HP segments. (7) BMOD C2d P13, quartz. Layer BMOD is above layer RSp and below OMOD. The BMOD morphology is found in layer MOD and in several of the Final MSA layers but is not common in older layers. Scale bar in cm.

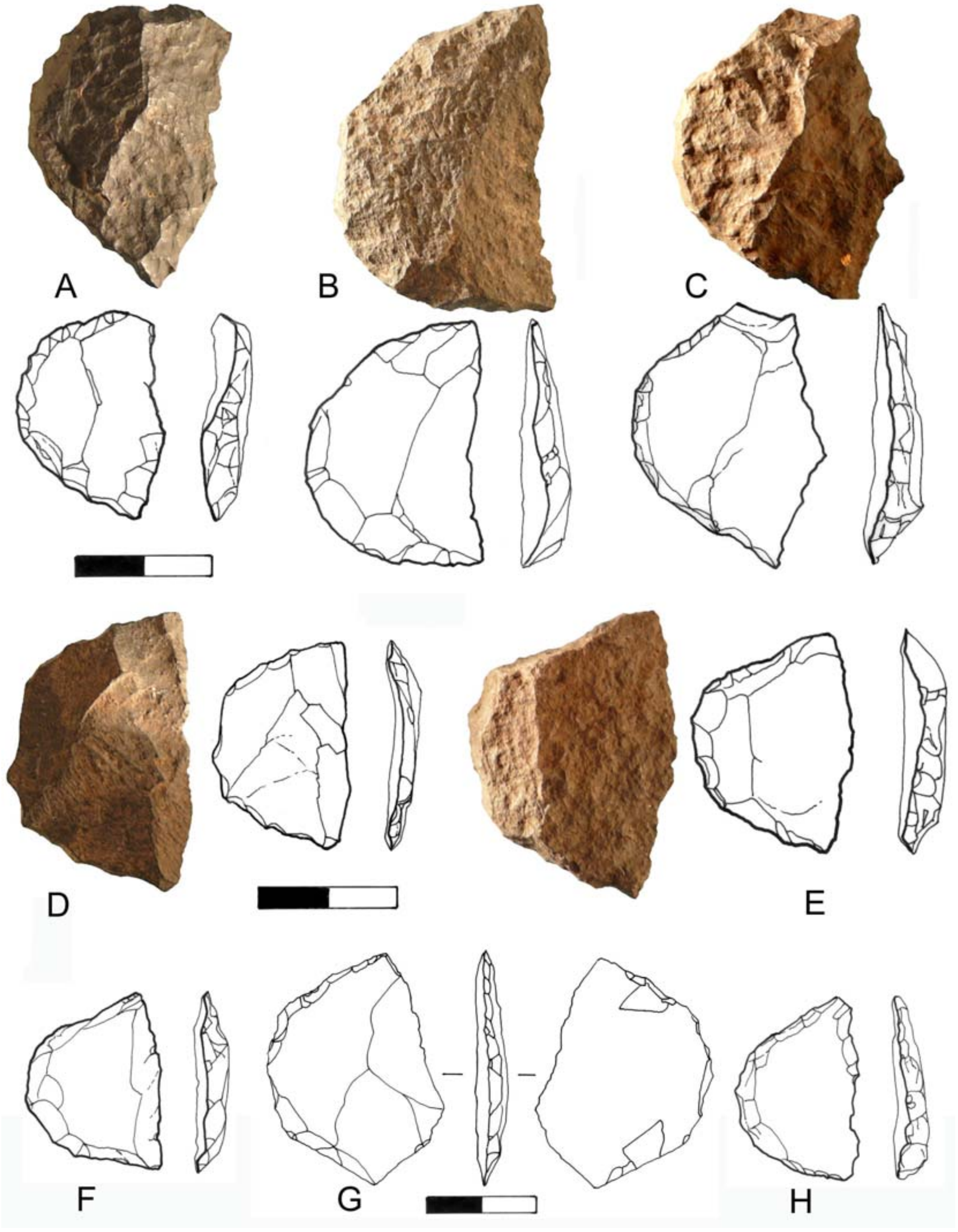

Fig. F. Backed pieces in the late-final MSA layers of Sibudu, dated c. 48-38 ka by OSL. All on flake and all of dolerite, except (D) of hornfels. (A) MOD square D6d no. 90. (B) LBMOD square D3c no. 31. (C) ES square C2a no. 5. (D) MC square E2a no. 1. (E) MC square D2a no. 5. (F) MC square E3b no. 3. (G) LBMOD square D3c no. 33. (H) LBMOD square D3a no. 32. Scale bar in cm.

References

1. Villa P, Delagnes A,Wadley L. A late Middle Stone Age artifact assemblage from Sibudu (KwaZulu- Natal). Comparisons with the European Middle Paleolithic. J Archaeol Sci. 2005; 32: 399-422.

2. Soriano S. Villa P, Wadley L. Blade technology and tool forms in the Middle Stone Age of South Africa: The Howiesons Poort and post-Howiesons Poort at Rose Cottage Cave. J Archaeol Sci. 2007; 34: 681–703.

3. Villa P, Soressi M, Henshilwood CS, Mourre V. The Still Bay points of Blombos Cave (South Africa). J Archaeol Sci. 2009; 36: 441-460.

4. Villa P, Soriano S, Teyssandier N, Wurz S. The Howiesons Poort and MSA III at Klasies River main site, Cave 1A. J Archaeol Sci. 2010**;** 37: 630–655.

5. Villa P**,** Soriano S,Tsanova T, Degano I, Higham TFG, d’Errico F, Backwell L, Lucejko JJ, Colombini MP, Beaumont PB. Border Cave and the beginning of the Later Stone Age in South Africa. Proc Natl Acad Sci USA. 2012**;** 109 (33): 13208-13213.

6. Wadley L, Kempson H. A review of rock studies for archaeologists, and an analysis of dolerite and hornfels from the Sibudu area, KwaZulu-Natal. South Afr Humanit. 2011; 23: 87-107.

7. Inizan ML, Tixier J. Outrepassage intentionnel sur pièces bifaciales néolithiques du Qatar (Golfe arabo-persique). Quaternaria. 1978; XX: 29-40.

8. Shea JJ. The origins of lithic projectile technology: evidence from Africa, the Levant, and Europe. J Archaeol Sci. 2006; 33: 823-846.

9. Sisk ML, Shea JJ. The African origin of complex projectile technology: an analysis using tip cross-sectional area and perimeter. Int J Evol Biol. 2011:1-8.

10. Newman K, Moore WM. Ballistically anomalous stone projectile points in Australia. J Archaeol Sci. 2013; 40: 2614-2620.

11. Villa P, Lenoir M. Hunting weapons of the Middle Stone Age and the Middle Palaeolithic: spear points from Sibudu, Rose Cottage and Bouheben. South Afr Humanit. 2006; 18: 89-122.

12. Villa P, Lenoir M. Hunting and hunting weapons of the Lower and Middle Paleolithic of Europe. In in. M. Richards M, Hublin JJ, editors. The Evolution of Hominin Diets. Springer, Dordrecht; 2009, pp. 59-84.

13. Will M, Bader GD, Conard NJ. Characterizing the Late Pleistocene MSA lithic technology of Sibudu, KwaZulu-Natal, South Africa. PloS One. 2014; 9: e398359.

14. Jacobs Z, Wintle AG, Duller GAT, Roberts RG, Wadley L.New ages for the post-Howiesons Poort, late and final Middle Stone Age at Sibudu, South Africa. J Archaeol Sci. 2008; 35: 1790-1807.
