# Supplementary material for: A Milk and Ochre Paint Mixture Used 49,000 Years Ago at Sibudu, South Africa
Source: PLoS One. 2015 Jun 30;10(6):e0131273. doi: 10.1371/journal.pone.0131273 (PMC4488428; doi:10.1371/journal.pone.0131273)
Supplement: S3 Text — (PDF) [file pone.0131273.s003.pdf]

Supporting Information

A milk and ochre paint mixture used 49,000 years ago at Sibudu, South Africa

Paola Villa, Luca Pollarolo, Ilaria Degano, Leila Birolo, Marco Pasero, Cristian Biagioni, Katerina Douka, Roberto Vinciguerra, Jeannette J. Lucejko, Lyn Wadley

S3 Text. Direct dating of the residue

Radiocarbon measurement was performed at the Oxford Radiocarbon Accelerator Unit (ORAU), University of Oxford, UK. We attempted direct dating of the flake residue twice. Initially, about 5mg of material still attached to the flake (P no. 37438.0) was weighed into a clean tin capsule and underwent combustion in a EA-CF-IRMS system, consisting of a elemental analyzer coupled to a gas source isotope ratio mass spectrometer. The C yield from this first combustion was low, ~100µg, which is about 1/8th of what is required for a reliable AMS determination at Oxford. We did not proceed in graphitization and AMS measurement of this fraction, and the sample was failed.

Given the non-homogenus composition of the residue and its potential for differential presence of C, we attempted to date the residue again. All visible remaining organic material was scraped of the tool using a clean scalpel and was placed in a tin capsule. About 13mg was collected this time (P no. 37438.1). The residue did not undergo any chemical pretreatment normally performed to purify endogenous C and eliminate any externally-derived C substances. Instead, it underwent direct combustion on the EA-IRMS system along with routinely measured standards (^{14}C-free charcoal). Although the lack of chemical cleaning meant that any contaminants would remain intact contributing to the overall ^{14}C determination, the decision was imposed by the very small sample size, its composition and the low C content we established previously (~2.3–3%). This second time, we obtained 390µg of C in the form of CO_2. The gas was graphitized using the routine methods of the ORAU (catalytic reaction in the presence of Fe and H in the ratio of 2.2 H_2:CO_2, at 560°C for 6h) and pressed into target holders prior to being introduced to the AMS.

The stable isotope values obtained during combustion are shown on Table A. The δ^{13}C was measured separately for each fraction (P. 37438.0 and 37.438.1), and, in both cases, it was identical (-20.7 to -20.6 ‰). While the value reflects a mixture of all C sources present in the residue, it is consistent with values expected from collagenous material derived from herbivores. The d^{15}N on the other hand is extremely high, and certainly unreliable due to the very small N amount detected in the sample.

The AMS ^{14}C determination for the residue is 23,350 ± 400 BP (OxA-X-2605-35) (uncalibrated radiocarbon years before present).

The lack of chemical cleaning and purification of the sample prior to dating let us suggest that OxA-X-2605-35 is a minimum age only and comes with a serious "health warning". The large standard error associated with the measurement is due to the small amount of C (390µg), about 5 times smaller than the "normal size" graphites routinely

measured at the ORAU and half in size of the ORAU's "small size" graphites, the cut-off being at ~800µg of C.

The significance of this direct measurement lies in the fact that it establishes the antiquity of the use of milk well before the introduction of domestic cattle in S. Africa in the 1st millennium AD.

Sample ID (P no.)	Used (mg)	mg C	mg N	$d^{13}C$ (‰)	$d^{15}N$ (‰)	^{14}C (yrs BP)	±	$F^{14}C$	±
37438.0	4.71	0.108	0.029	-20.7	n/a	Failed due to low yield			
37438.1	13	0.390	0.088	-20.6	24.12*	**23350**	**400**	0.0548	0.0027

Table A. Results of the radiocarbon mesurements. The age obtained is a minimum age only due to the overwhelming siginificance of modern carbon contamination on ancient material.
