# Supplementary material for: A Milk and Ochre Paint Mixture Used 49,000 Years Ago at Sibudu, South Africa
Source: PLoS One. 2015 Jun 30;10(6):e0131273. doi: 10.1371/journal.pone.0131273 (PMC4488428; doi:10.1371/journal.pone.0131273)
Supplement: S4 Text — (PDF) [file pone.0131273.s004.pdf]

Supporting Information

A milk and ochre paint mixture used 49,000 years ago at Sibudu, South Africa

Paola Villa, Luca Pollarolo, Ilaria Degano, Leila Birolo, Marco Pasero, Cristian Biagioni, Katerina Douka, Roberto Vinciguerra, Jeannette J. Lucejko, Lyn Wadley

S4 Text. Chemical analyses

Gas chromatography/mass spectrometry (GC/MS) of the MOD flake

Micro-samples (3.3 mg from the MOD flake and less than 1 mg from the other samples, see below) were collected from the specimens and submitted to an analytical procedure (GC/MS) in order to identify lipids, waxes, proteins, resinous materials and saccharides [1-2]. GC/MS had already been successfully employed for the characterization of organic materials from Border Cave [3].

Reagents

All the solvents were Baker HPLC grade and were used without any further purification. Trifluoroacetic acid (99% purity) and anhydrous pyridine were from Fluka (Milan, Italy). Ethanethiol (ETSH; 99.5%), sodium azide (NaN3; 99.5%), *N*,*O*-bis(trimethylsilyl)trifluoroacetamide (BSTFA) with and without 1% trimethylchlorosilane, *N*-*tert*-butyldimethylsilyl-*N*methyltrifluoroacetamide (MTBSTFA) with 1% trimethylchlorosilane, 1,1,1,3,3,3-hexamethyldisilazane (HMDS, 99.9%) and triethylamine were from Sigma-Aldrich. The following solutions, apart from those for the amino acids, were prepared by weighing pure substances and were used as standards: (i) amino acid solution in 0.1 M HCl (Sigma-Aldrich) and containing 12.5 μmol/mL of proline (Pro) and hydroxyproline (Hyp) and 2.5 μmol/mL of aspartic acid (Asp), glutamic acid (Glu), alanine (Ala), arginine, cysteine, phenylalanine (Phe), glycine (Gly), hydroxylysine, isoleucine (Ile), histidine, leucine (Leu), lysine (Lys), methionine (Met), serine (Ser), tyrosine (Tyr), threonine, and valine (Val); (ii) solution of fatty and dicarboxylic acids in acetone, containing lauric acid (0.24 mg/ g), suberic acid (0.27 mg/g of Su), azelaic acid (0.28 mg/g of A), myristic acid (0.25 mg/g of My), sebacic acid (0.3 mg/g of Se), palmitic acid (0.25 mg/g of P), oleic acid (0.51 mg/g of O), stearic acid (0.51 mg/g of S) [all acids (purity >99%) from Sigma-Aldrich]; (iii) norleucine solution in bidistilled water (Sigma-Aldrich; purity 99%, 138.66 μg/g) was used as a derivatization internal standard for amino acids; (iv) tridecanoic acid solution in isooctane (Sigma-Aldrich; purity 99%, 135.48 μg/g) was used as a lipid-resinous fraction derivatization internal standard; (v) hexadecane solution in isooctane (Sigma-Aldrich; purity 99%, 80.34 μg/g) was used as an injection internal standard; (vi) monosaccharides and uronic acids solution in bidistilled water containing D-(+)-galactose (0.1 mg/g), L-(-)-fucose (0.1 mg/g), L-(+)-arabinose (0.1 mg/g), L-(-)-ramnose (0.1 mg/g), L-(-)-mannose (0.1 mg/g), D-(+)-xylose (0.1 mg/g), D-(+)-glucose (0.1 mg/g), D-glucuronic

acid (0.1 mg/g), D-galacturonic acid (0.1 mg/ g) monohydrate; and (xi) mannitol in bidistilled water (0.1 mg/g) was used as a derivatisation internal standard for aldoses and uronic acids. All monosaccharides and uronic acids (purity 99%) were purchased from Sigma-Aldrich (Milan, Italy). All standard solutions were stored at 4 °C.

Analytical procedure and instrumentation

The sample treatment, needed prior to GC/MS analysis, consists in a multistep chemical pre-treatment based on the ammonia extraction of proteins and polysaccharide materials, in order to separate them from lipid and resinous materials. The extraction is then followed by the separation and purification of proteinaceous and polysaccharide materials before hydrolysis. Lipids and resins are saponified/salified separately. Three fractions are generated, derivatised with silylating agents and analyzed separately by GC/MS, thus enabling a quantitative analysis to be performed.

The acidic hydrolysis of proteins and gums and the saponification of lipids were performed using a microwave oven model ETHOS One (High Performance Microwave Digestion System) Milestone (Sorisole, Bergamo, Italy). The GC/MS instrumentation consists of a 6890N Network GC System (Agilent Technologies, Palo Alto, CA, USA) equipped with a PTV injector and coupled to a 5973 MS detector with quadrupole analyser. MS was set with an electron impact ionisation (EI, 70 eV) in positive mode, ion source temperature at 230 °C, scan range of 50-700 m/z, and interface temperature of 280 °C. GC separation was performed using a HP-5MS column (J&W Scientific, Agilent Technologies: stationary phase 5% phenil–95% methylpolysiloxane, 30 m length, 0.25 mm i.d., 0.25 m film thickness) connected to a deactivated fused silica precolumn (J&W Scientific, Agilent Technologies: 2 m length, 0.32 mm i.d.). GC conditions for the lipid-resinous fraction involved the use of the PTV injector in splitless mode at 300 °C; the chromatographic oven was programmed at 80 °C for 2 min isothermal, 10 °C/min up to 200 °C, 4 min isothermal, 6 °C/min up to 280 °C, 40 minutes isothermal; constant He flow 1.2 mL/min, injector temperature 280 °C. GC conditions for the protein fraction involved the PTV injector used in splitless mode at 220 °C; the chromatographic oven was programmed with an initial temperature of 100 °C, isothermal for 2 min, then 4°C/min up to 280 °C, and 280 °C isothermal for 15 min. GC conditions for the saccharide fraction involved the PTV injector used in splitless mode at 250 °C; the chromatographic oven was programmed at 50 °C isothermal for 2 min, 5°C/min up to 190 °C, 190 °C isothermal for 20 min 5 °C/min up to 280 °C, and 280 °C isothermal for 15 min. Structural assignments are based on literature data, NIST and Wiley mass spectra libraries and spectra interpretation.

Analysis of sample D6d MOD: results

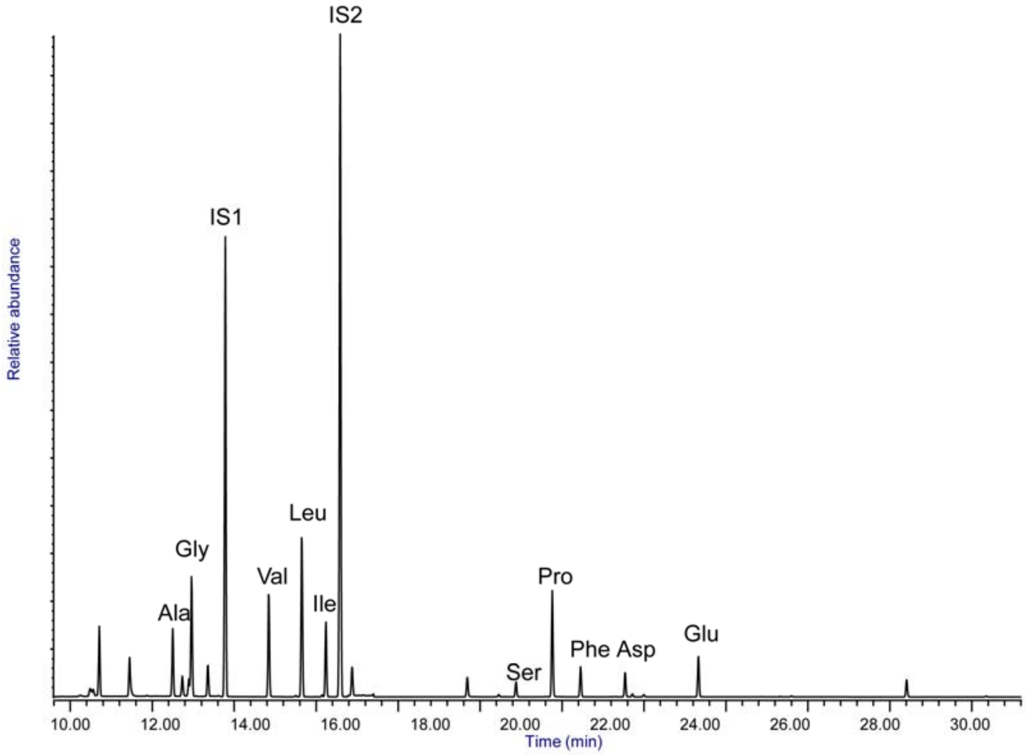

Fig. A. Gas chromatogram of the proteinaceous fraction of sample D6 MOD acquired in SIM mode (Ala = alanine, Gly = glycine, Val= valine, Leu = leucine, Ile = isoleucine, Pro = proline, Phe = phenylalanine, Asp = aspartic acid, Glu = glutammic acid, Hyp = hydroxyproline, IS1 = hexadecane, IS2 = norleucine).

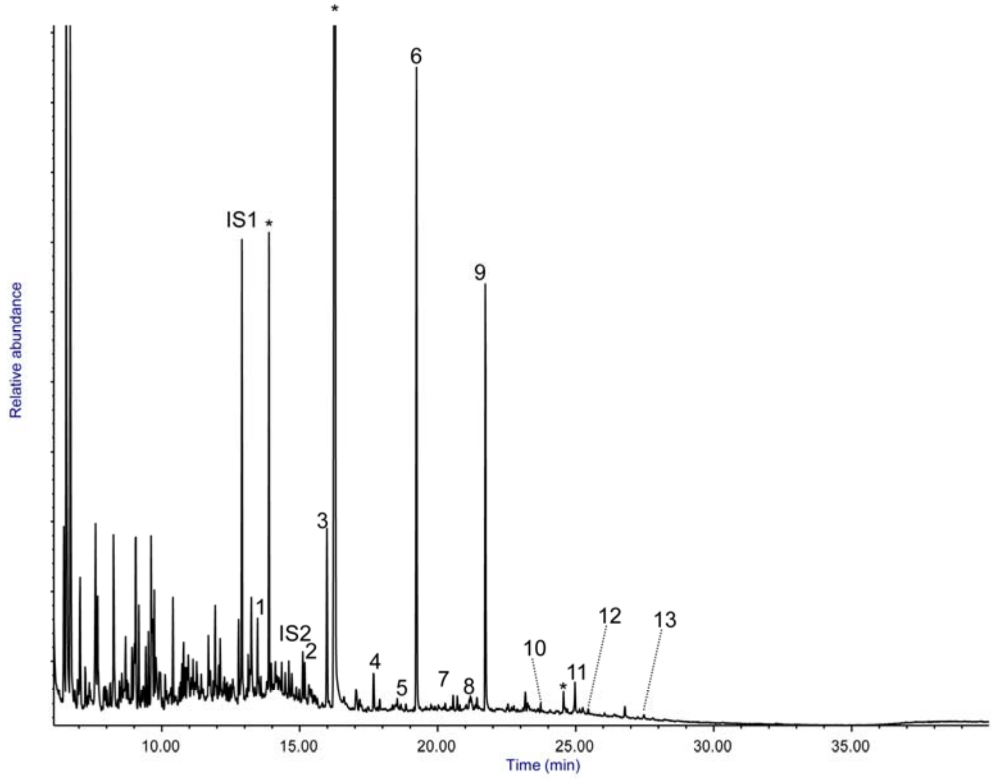

Fig. B. Gas chromatogram of the lipid-resinous fraction of the MOD flake residue (IS1 = hexadecane, IS2 = tridecanoic acid). The peak assignment is given in Table A.

Table A. Peak assignment for Fig. B. Acids and alcohols are identified as their trimethylsilyl (TMS) derivatives.

Peak n°	Retention time (min)	Identified compound
1	13.85	dodecanoic acid (lauric)
2	15.65	α,ω-nonanedioc acid (azelaic)
3	16.54	tetradecanoic acid (myristic)
4	18.24	pentadecanoic acid (also branched)
5	18.83	cis-9-hexadecenoic acid (palmitoleic)
6	19.72	hexadecanoic acid (palmitic)
7	21.02	heptadecanoic acid (also branched)
8	21.42	cis-9-octadecenoic acid (oleic)
9	21.74	octadecanoic acid (stearic)
10	23.73	eicosanoic acid
11	24.97	unknown peak (m/z 217, 315, 371, 463)
12	25.46	docosanoic acid
13	27.48	tetracosanoic acid

GC/MS analysis of samples of Howiesons Poort backed segments and other tools

Six stone artifacts with ochre lines and possible residue in the hafting area have been analyzed using the same procedures applied to the MOD flake. The samples are:

- the backed edge of three Howiesons Poort segments from Sibudu (GR C6c, PGS B5a and GSII B6a, dated between 64.7 ± 2.3 and 61.7 ± 2.0 [4]; Fig. C;
- the backed edge of one segment from Rose Cottage (MAL IH no. 16; described with residues in [5: fig. 2:4);
- the base of a unifacial point in layer Co (C3a CO, final MSA, dated to 38.0 ± 2.6 ka by OSL [6];
- the thick back of a side scraper (from MOD C3a).

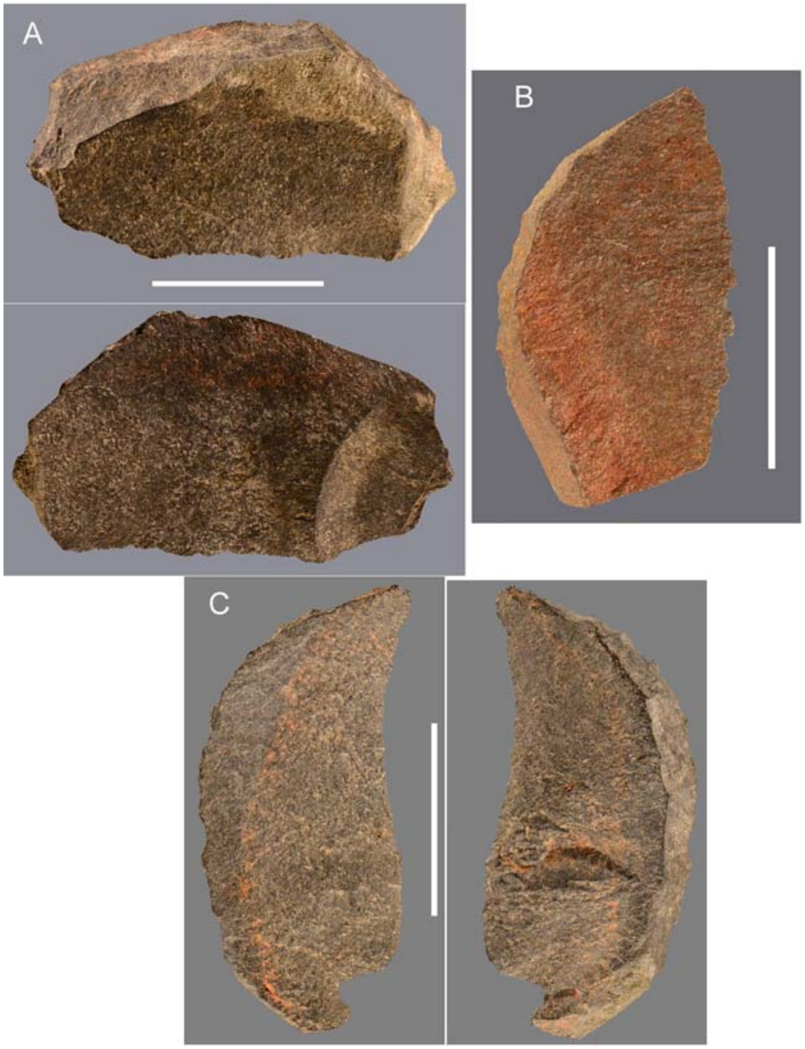

Fig. C. Three backed pieces from the Howiesons Poort layers of Sibudu, all hornfels. (A) PGS B5a no. 69. (B) GR C6c, broken at base. (C) GSII B6a. All three were dipped in solvent prior to analysis but only A and B gave significant results. Scale bar = 1 cm.

All six samples were lightly scraped in the described area. No significant molecular markers of organic material were detected in four samples. Three backed segments, PGS B5a, GR C6c and GSII B6a were then dipped in the extraction solvent for the first step of the combined procedure. Two gave significant results illustrated by Figs. D-E for PGS B5a and GR C6c, respectively. Peak assignment is reported in Table B.

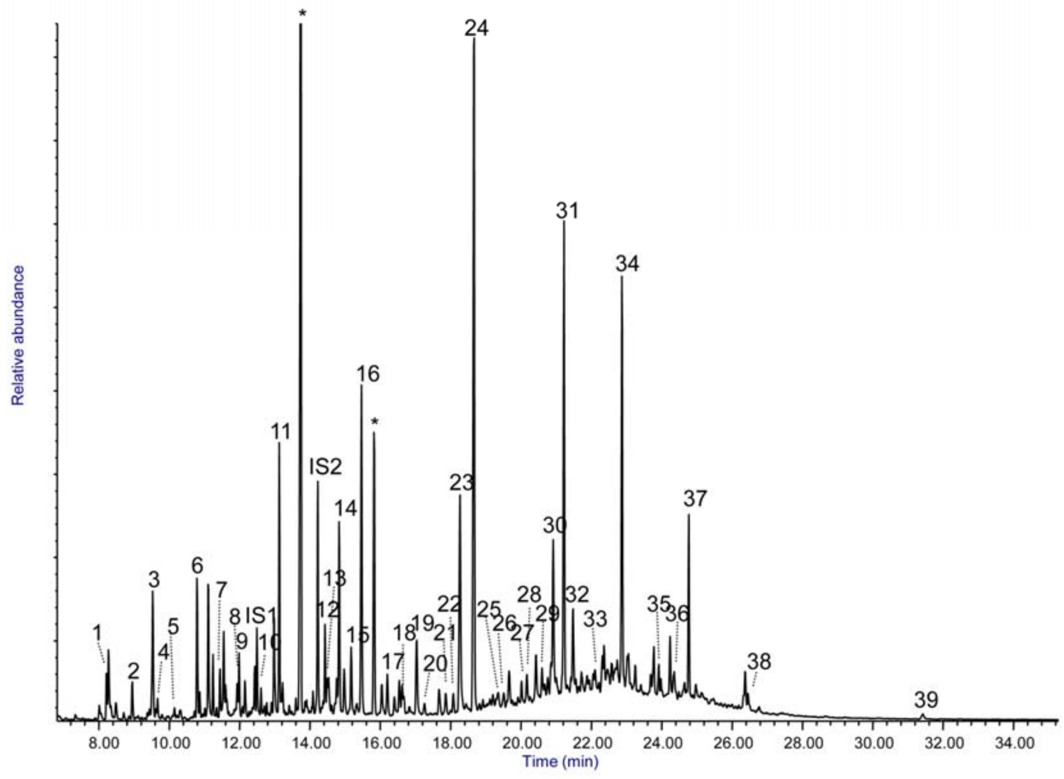

Fig. D. Gas chromatogram of the total lipid-resinous fraction of sample B5a PGS (IS1 = hexadecane, IS2 = tridecanoic acid). Peak assignment is reported in Table B.

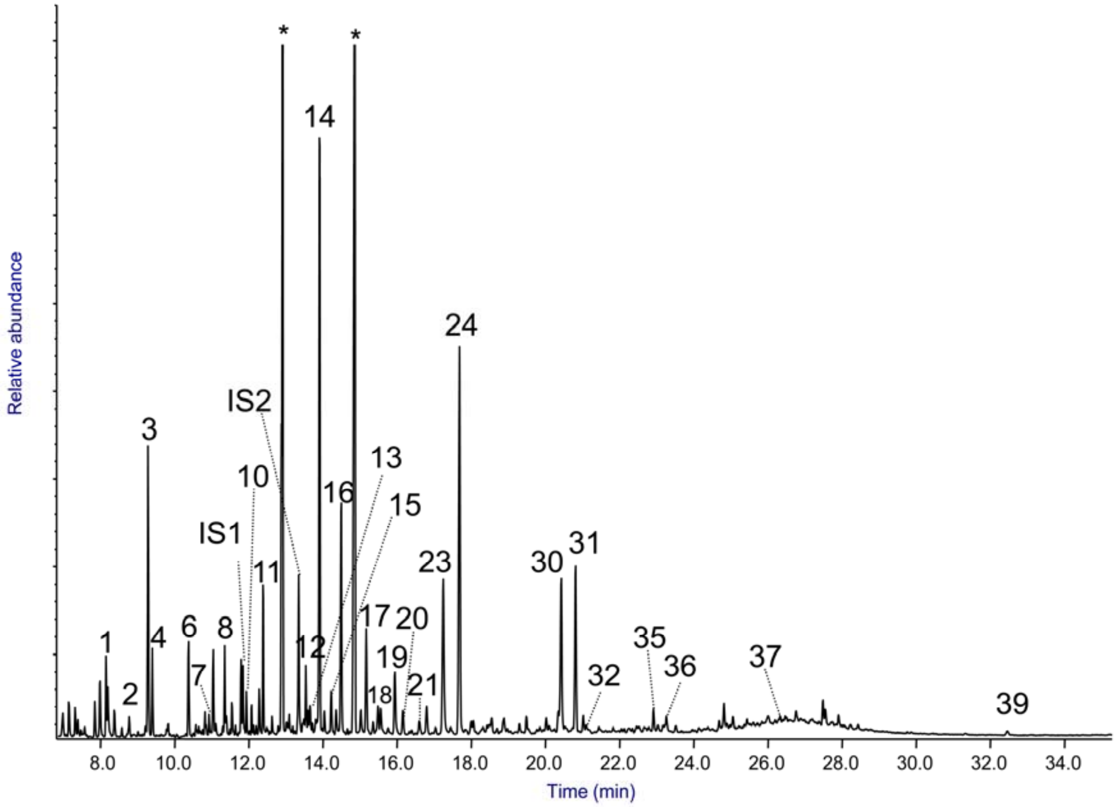

Fig. E. Gas chromatogram of the total lipid-resinous fraction of sample C5c GR (IS1 = hexadecane, IS2 = tridecanoic acid). Peak assignment is reported in Table B.

Table B. Peak assignment for figs. D-E.

Peak #	Tr (min)	Compound	Peak #	Tr (min)	Compound
1	8.29	octanoic acid	20	17.26	hexadecanol
2	8.95	succinic acid	21	17.85	undecandioic acid
3	9.53	nonanoic acid	22	18.08	12-hydroxydodecanoic acid
4	9.68	2-hydroxy heptanoic acid	23	18.27	cis-9-hexadecenoic acid (palmitoleic)
5	10.15	pentanedioic acid (glutaric acid)	24	18.66	hexadecanoic acid (palmitic)
6	10.80	decanoic acid	25	19.35	dodecandioic acid
7	11.44	hexanedioic acid (adipic acid)	26	19.51	13-hydroxytridecanoic acid
8	11.93	2-hydroxy nonanoic acid	27	20.00	heptadecanoic acid (also branched)
9	11.98	undecanoic acid	28	20.17	octadecanol (stearyl alcohol)
IS1	12.49	hexadecane	29	20.61	tridecanedioic acid
10	12.62	heptanedioic acid	30	20.91	cis-9-octadecenoic acid (oleic)
11	13.13	dodecanoic acid (lauric)	31	21.20	octadecanoic acid (stearic)
IS2	14.22	tridecanoic acid	32	21.48	isopimaric acid
12	14.43	p-hydroxy hydrocinnamic acid	33	22.10	16-hydroxyhexadecanoic acid
13	14.48	vanillic acid	34	22.86	dehydroabietic acid
14	14.83	nonandienoic acid (azelaic acid)	35	23.92	didehydroabietic acid
15	15.17	myristoleic acid (9-tetradecenoic acid)	36	24.34	9,10-dihydroxyoctadecanoic acid
16	15.46	tetradecanoic acid (myristic)	37	24.76	7-oxo-dehydroabietic acid
17	16.20	decanedioic acid (sebacic acid)	38	26.45	15-hydroxy-7-oxodehydroabietic acid
18	16.62	pentadecenoic acid	39	31.43	Cholesterol
19	16.51	pentadecanoic acid (also branched)			

The most abundant compounds contained in samples B5a PGS and C6c GR are linear monocarboxylic fatty acids (from C8 to C18), the most abundant being palmitic (hexadecanoic acid, C16:0) and stearic acids (octadecanoic, C18:0). The samples also contain dicarboxylic acids (in particular azelaic acid – nonandioic acid). These oxidation products of unsaturated fatty acids indicate that the lipids originally present in the samples contained a relevant amount of oleic acid (9-octadecanoic), which has undergone a degradative oxidation process, which is also revealed by the presence of 9,10-hydroxy-octadecanoic acids. The presence of odd chain length fatty acids and cholesterol indicates animal origin of fats. Odd-branched (C15 and C17) fatty acids suggest animal fats, or that the lipids have undergone bacterial degradation. On the basis of the observed lipid profile, it is not possible to say whether a vegetal-origin lipid material had been added to the mixture or not. In both samples, a diterpenic fraction was present, whose main

components are abietadienic acids: dehydroabietic (#34) , didehydroabietic (#35), 7-oxo-dehydroabietic (#37) and 15-hydroxy-7-oxodehydroabietic (#38). Pimaradienic acids such peak #32 (isopimaric acid) were also detected (pimaric, sandaracopimaric, palustric, levopimaric acids were detected as minor components). The diterpenic profile of the sample suggests that the hafting material contained a resin extracted from a plant belonging to the conifer family. In particular, *Pinaceae*, *Podocarpaceae* or *Araucariaceae* may produce a resin containing abietadienic and pimaradienic components. Amongst our database of South African plants, the wood of *Afrocarpus* (syn. *Podocarpus*) *falcatus* contains relevant amount of these compounds and is thus a probable candidate as the source of the resin used as hafting material. Analysis of charcoal from the HP layers of Sibudu shows that *Podocarpus* was predominant while *Acacia* is absent (7). The hypothesized use of plant gum as a hafting adhesive at Sibudu requires further testing.

Proteomic analyses

Proteinaceous material was identified on the MOD flake following a minimally invasive proteomic analytical procedure described by [8], aimed at preserving the integrity of the archeological piece. The micro-black sample n°87 of ca. 4 mg was directly submerged in 100 µl ammonium bicarbonate 10 mM pH 8.0, and trypsin digested in heterogeneous phase enzymatic by adding proteomics-grade trypsin 0.1µg/µl at 37 °C for 16 h. The supernatant was then recovered by centrifugation and the peptide mixture was filtered on 0.22 µm PVDF membrane (Millipore), concentrated and purified using a reverse-phase C18 Zip Tip pipette tip (Millipore). The eluate was analyzed by LC-MSMS on a 6520 Accurate-Mass Q-Tof LC/MS System (Agilent Technologies, Palo Alto, CA, USA) equipped with a 1200 HPLC System and a chip cube (Agilent Technologies). After loading, the peptide mixture was first concentrated and washed on a 40 nl enrichment column (Agilent Technologies chip), with 0.1% formic acid in 2% acetonitrile as eluent. The sample was then fractionated on a C18 reverse-phase capillary column (Agilent Technologies chip) at a flow rate of 400nL/min, with a linear gradient of eluent B (0.1% formic acid in 95% acenonitrile) in A (0.1% formic acid in 2% acetonitrile) from 7% to 80% in 50min. Peptide analysis was performed using data-dependent acquisition of one MS scan (mass range from 300 to 2000 m/z) followed by MS/MS scans of the three most abundant ions in each MS scan. MS/MS spectra were measured automatically when the MS signal surpassed the threshold of 50,000 counts. Double and triple charged ions were preferably isolated and fragmented. Each LC-MSMS analysis was preceded and followed by blank runs to avoid carryover contamination. The acquired MS/MS spectra were transformed in *mzData*(.XML) format and used for protein identification with a licensed version of MASCOT software (www.matrixscience.com) version 2.4.0.

Data handling. Preliminary data obtained by nanoLC-MSMS analysis were used to query the SwissProt database 2012_08 (537505 sequences; 190795142 residues), without any taxonomy restriction. Mascot search parameters were: trypsin as enzyme; 3, as allowed number of missed cleavage;10 ppm MS tolerance and 0.6 Da MSMS tolerance; peptide charge from +2 to +3. No fixed chemical modification was inserted, but possible oxidation of methionine and the formation of pyroglutammic acid from glutamine residues at the N-terminal position of peptides were considered as variable modification. As soon as collagen proteins were identified, a new identification run was carried out, with the insertion of hydroxylation on Lysine and Proline as variable modifications, since more confident identifications are commonly obtained with these proteins by taking into consideration their extensive post-translational modifications [8].

Individual ion score threshold provided by software to evaluate the quality of matches in MSMS data was 41. Spectra with score of <20, having low quality were rejected. Error tolerant searches were also carried out for each sample using as variable modifications proline and lysine hydroxylation to allow for single amino acid substitutions, enzyme non-specificity, as well as to check for unexpected modifications potentially induced by aging.

Bone samples. A set of four bovid size II and III [9] and one equine (*Equus quagga*) bone samples from Sibudu, layer MOD, were selected for the analysis (identifications by

Ina Plug, catalogue nos. 86, 91,1117, 1120, and 69). Approximately 3 mg of bone powder from each sample was weighed into a 1.5 mL microtube and demineralised, to get the highest sequence coverage possible for these reference collagens,with extensive incubation in 400 µl 0.5 M EDTA pH 8 at 4°C for 24 h. Insoluble pellets were then extensively washed with MilliQ water, and then submerged in 200 µl ammonium bicarbonate 10 mM pH 8.0, and trypsin digested in heterogeneous phase as described above for the MOD flake.

Table C. Identification of the proteins in the MOD sample from Sibudu by overnight trypsin digestion in heterogeneous phase and LCMSMS analysis[a]. Proteins were identified searching UniprotSprot database, without any taxonomy restriction, with MS/MS Ion search Mascot software (Matrix Science).

**Identified protein** (Accession number)	**Total score[a]**	**Sequence coverage** (%)	**Individual ion score**	**Matched sequence** (Oxidation of methionine, hydroxylation of proline and lysine, pyro-Glu formation at Gln at the N-terminus of peptides were inserted as variable modifications).
			20	**GAPGPAGPK** + Hydroxy (P)
			26	**GAAGLPGPK**+ Hydroxy (P)
			34	**GPAGPQGPR**
			27	**GFSGLDGAK**
			44	**GSEGPQGVR**
			26	**GFPGADGVAGPK** + Hydroxy (P)
			33	**GADGAPGKDGVR**
			29	**GVQGPPGPAGPR** + Hydroxy (P)
			28	**GQAGVMGFPGPK**+ Oxidation (M); Hydroxy (K)
			40	**GLTGSPGSPGPDGK**+ 2 Hydroxy (P)
			24	**GFPGLPGPSGEPGK** + Hydroxy (P)
			26	**GPSGPQGPSGPPGPK** + Hydroxy (P)
			24	**GETGPAGPAGPIGPVGAR**
			21	**GSPGEAGRPGEAGLPGAK** + Hydroxy (K); 2 Hydroxy (P)
			20	**EGAPGAEGSPGRDGSPGAK** + Hydroxy (K); 2 Hydroxy (P)
**Collagen alpha-1(I) chain** (P02453)	**832**	**40**	22	**GPPGPMGPPGLAGPPGESGR** + Oxidation (M); 2 Hydroxy (P)
			36	**GEPGPTGIQGPPGPAGEEGK** + 2 Hydroxy (P)
			31	**SGDRGETGPAGPAGPIGPVGAR**
			36	**GEPGPTGIQGPPGPAGEEGKR** + 2 Hydroxy (P)
			41	**GDRGETGPAGPPGAPGAPGAPGPVGPAGK** + 4 Hydroxy (P)
			26	**GAPGDRGEPGPPGPAGFAGPPGADGQPGAK**+ 4 Hydroxy (P)
			58	**GFSGLQGPPGPPGSPGEQGPSGASGPAGPR** + 3 Hydroxy (P)
			29	**GLTGPIGPPGPAGAPGDKGEAGPSGPAGPTGAR** + Hydroxy (K); Hydroxy (P)
			35	**GLPGADGR** + Hydroxy (P)
			21	**GATGPAGVR**
			26	**GVVGPQGAR**
			46	**VGAPGPAGAR**+ Hydroxy (P)
			24	**GEPGNIGFPGPK** + 2 Hydroxy (P)
			36	**GPAGPSGPAGKDGR**
			32	**GIPGEFGLPGPAGAR** + 2 Hydroxy (P)
**Collagen alpha-2(I) chain** (P02465)	**575**	**24**	21	**GDGGPPGATGFPGAAGR** + 2 Hydroxy (P)
			25	**GAAGLPGVAGAPGLPGPR**+ 3 Hydroxy (P)
			35	**GPPGESGAAGPTGPIGSR** + Hydroxy (P)
			30	**GSTGEIGPAGPPGPPGLR** + 2 Hydroxy (P)
			20	**EGPVGLPGIDGRPGPIGPAGAR+** 2 Hydroxy (P)
			65	**GLPGVAGSVGEPGPLGIAGPPGAR** + 3 Hydroxy (P)

**Alpha-S1-casein**<br>(P02662)	**249**	**33**	40<br>20<br>31<br>24<br>59	**EDVPSER**<br>**YLGYLEQLLR**<br>**FFVAPFPEVFGK**<br>**VPQLEIVPNSAEER**<br>**HQGLPQEVLNENLLR**
**Alpha-S2-casein**<br>(P02663)	**160**	**23**	34<br>30<br>28<br>28	**TKVIPYVR**<br>**FALPQYLK**<br>**NAVPITPTLNR**<br>**ALNEINQFYQK**
**Beta-casein**<br>(P02666)	**99**	**12**	24<br>25<br>21	**VLPVPQK**<br>**AVPYPQR**<br>**VLPVPQKAVPYPQR**
**Kappa-casein**<br>(P02668)	**47**	**14**	24<br>23	**YIPIQYVLSR**<br>**SPAQILQWQVLSNTVPAK**

Proteins were identified searching UniprotSprot database with MSMS Ion search Mascot software (Matrix Science) without any taxonomy restriction, with oxidation on Met, hydroxylation on Lys and Pro as variable modifications. Individual ion score above the significance threshold (>41) indicate identity or extensive homology (p<0.05). Only peptides with individual ion scores >20 were considered for identification purposes. Only proteins identified with at least two peptides were considered as significant.

```
1    MKLLILTCLVAVALARPKHPIKHQGLPQEVLNENLLRFFVAPFPEVFGKEKVNEL-----   55  P02662   CASA1_BOVIN
1    MRLLILTCLVAVALARPKLPLRYPERLQNPSE------SSEPIPLESREEYMNGMNRQRN   54  P47710   CASA1_HUMAN
1    MKLLILTCLVAVALARPKHPINHRGLSPEVPNENLLRFVVAPFPEVFRKENINEL-----   55  P18626   CASA1_CAPHI
1    MKLLILTCLVAVALARPKHPIKHQGLSSEVLNENLLRFVVAPFPEVFRKENINEL-----   55  P04653   CASA1_SHEEP
     *:**************** *:.:     :  :         *:*    :* :* :

56   ---------SKDIGSESTEDQAMEDIKQMEAESISSSEEIVPNSVEQKHIQKEDVPSERY  106  P02662   CASA1_BOVIN
55   ILREKQTDEIKDTRNESTQNCVVAEPEKMESSISSSSEEMS-----------------L   96  P47710   CASA1_HUMAN
56   ---------SKDIGSESTEDQAMEDAKQMKAGSSSSSEEIVPNSAEQKYIQKEDVPSERY  106  P18626   CASA1_CAPHI
56   ---------SKDIGSESIEDQAMEDAKQMKAGSSSSSEEIVPNSAEQKYIQKEDVPSERY  106  P04653   CASA1_SHEEP
               **  .** :: .: : ::*::   *****:

107  LGYLEQLLRLKKYKVPQLEIVPNSAEERLHSMKEGIHAQQKEPMIGVNQELAYFYPELFR  166  P02662   CASA1_BOVIN
97   SKCAEQFCRLNEYNQLQLQ--AAHAQEQIRRMNENSH-----------------VQVPFQ  137  P47710   CASA1_HUMAN
107  LGYLEQLLRLKKYNVPQLEIVPKSAEEQLHSMKEGNPAHQKQPMIAVNQELAYFYPQLFR  166  P18626   CASA1_CAPHI
107  LGYLEQLLRLKKYNVPQLEIVPKSAEEQLHSMKEGNPAHQKQPMIAVNQELAYFYPQLFR  166  P04653   CASA1_SHEEP
         **: **::*:  **:     *:*::: *:*                        *:

167  QFYQLDAYPSGAWYYVPLGTQYTDAPSFSDIPNPIGSENSEKTT-MPLW  214  P02662   CASA1_BOVIN
138  QLNQLAAYPYAVWYYPQ-IMQYVPFPPFSDISNPTAHENYEKNNVMLQW  185  P47710   CASA1_HUMAN
167  QFYQLDAYPSGAWYYLPLGTQYTDAPSFSDIPNPIGSENSGKTT-MPLW  214  P18626   CASA1_CAPHI
167  QFYQLDAYPSGAWYYLPLGTQYTDAPSFSDIPNPIGSENSGKIT-MPLW  214  P04653   CASA1_SHEEP
     *: ** *** ..***     **.  * **** ** . **  * . *  *
```

Fig. F. Alignment of Alpha-S1-casein sequences from cattle (*Bos taurus*, P02662), human (*Homo sapiens*, P47710), goat (*Capra hircus*, P18626), and sheep (*Ovis aries*, P04653). Highlighted in grey and bold are the matched sequences.

Fig. G . Alignment of the matched peptides in the MOD flake to the Collagen alpha-1(I) chain from *Bos taurus* (P02453) and *Equus asinus* (B9VR88). Peptides identified in the MOD flake sample were manually aligned over the automatically generated alignment (CLUSTAL O(1.2.0) multiple sequence alignment, of the two collagen alpha-1(I) chain from *Bos taurus* (P02453) and *Equus asinus* (B9VR88). Peptides from MOD flake are reported in bold. An * (asterisk) indicates positions which have a single, fully conserved residue; : (colon) indicates conservation between groups of strongly similar properties; . (period) indicates conservation between groups of weakly similar properties. Under the alignment, peptides matched in samples from bones of some Bovids and *Equus quagga* remains recovered from Sibudu layer MOD, are reported. Underlined sequences are those identified in the error tolerant database search and the modified residue is indicated in red. Numbering refers to the precursor polypeptide chain while only the sequence of the mature protein is reported.

```
MOD flake

P02453 Bos taurus                                            QLSYGYDEKSTGISVPGPM 180
B9VR88 Equus asinus                                          QLSYGYDEKSAGISVPGPM 180
                                                             **********:********

#86   Bovid                                                           STGISVPGPM
#69   Equus quagga
#1120 Bovid
#1117 Bovid                                                           STGISVPGPM
#91   Bovid


MOD flake

P02453 Bos taurus     GPSGPRGLPGPPGAPGPQGFQGPPGEPGEPGASGPMGPRGPPGPPGKNGDDGEAGKPGRP 240
B9VR88 Equus asinus   GPSGPRGLPGPPGAPGPQGFQGPPGEPGEPGASGPMGPRGPPGPPGKNGDDGEAGKPGRP 240
                      ************************************************************

#86   Bovid           GPSGPRGLPGPPGAPGPQGFQGPPGEPGEPGASGPMGPRGPPGPPGKNGDDGEAGKPGRP
#69   Equus quagga                                           GPPGPPGKNGDDGEAGKPGRP
#1120 Bovid                                                     GPPGPPGK
#1117 Bovid           GPSGPRGLPGPPGAPGPQGFQGPPGEPGEPGASGPMGPRGPPGPPGKNGDDGEAGKPGRP
#91   Bovid                                                  GPPGPPGK


MOD flake                                        GFSGLDGAK

P02453 Bos taurus     GERGPPGPQGARGLPGTAGLPGMKGHRGFSGLDGAKGDAGPAGPKGEPGSPGENGAPGQM 300
B9VR88 Equus asinus   GERGPPGPQGARGLPGTAGLPGMKGHRGFSGLDGAKGDAGPAGPKGEPGSPGENGAPGQM 300
                      ************************************************************

#86   Bovid           GERGPPGPQGARGLPGTAGLPGMK   GFSGLDGAKGDAGPAGPKGEPGSPGENGAPGQM
#69   Equus quagga    GERGPPGPQGAR                                GEPGSPGENGAPGQM
#1120 Bovid              GPPGPQGAR               GFSGLDGAKGDAGPAGPKGEPGSPGENGAPGQM
#1117 Bovid           GERGPPGPQGARGLPGTAGLPGMK   GFSGLDGAKGDAGPAGPKGEPGSPGENGAPGQM
#91   Bovid              GPPGPQGAR


MOD flake

P02453 Bos taurus     GPRGLPGERGRPGAPGPAGARGNDGATGAAGPPGPTGPAGPPGFPGAVGAKGEGGPQGPR 360
B9VR88 Equus asinus   GPRGLPGERGRPGAPGPAGARGNDGATGAAGPPGPTGPAGPPGFPGAVGAKGEAGPQGAR 360
                      *****************************************************.**** *

#86   Bovid           GPRGLPGERGRPGAPGPAGARGNDGATGAAGPPGPTGPAGPPGFPGAVGAK
#69   Equus quagga    GPRGLPGERGRPGAPGPAGARGNDGATGAAGPPGPTGPAGPPGFPGAVGAKGEAGPQGAR
#1120 Bovid           GPRGLPGERGRPGAPGPAGAR
#1117 Bovid           GPRGLPGERGRPGAPGPAGARGNDGATGAAGPPGPTGPAGPPGFPGAVGAK
#91   Bovid                    GRPGAPGPAGARGNDGATGAAGPPGPTGPAGPPGFPGAVGAK
```

```
MOD flake               GSEGPQGVR                                         GFPGARGPSGPQ

P02453 Bos taurus       GSEGPQGVRGEPGPPGPAGAAGPAGNPGADGQPGAKGANGAPGIAGAPGFPGARGPSGPQ 420
B9VR88 Equus asinus     GSEGPQGVRGEPGPPGPAGAAGPAGNPGADGQPGAKGANGAPGIAGAPGFPGARGPSGPQ 420
                        ************************************************************

#86   Bovid             GSEGPQGVRGEPGPPGPAGAAGPAGNPGADGQPGAKGANGAPGIAGAPGFPGARGPSGPQ
#69   Equus quagga      GSEGPQGVRGEPGPPGPAGAAGPAGNPGADGQPGAKGANGAPGIAGAPGFPGARGPSGPQ
#1120 Bovid             GSEGPQGVRGEPGPPGPAGAAGPAGNPGADGQPGAKGANGAPGIAGAPGFPGARGPSGPQ
#1117 Bovid             GSEGPQGVRGEPGPPGPAGAAGPAGNPGADGQPGAKGANGAPGIAGAPGFPGARGPSGPQ
#91   Bovid             GSEGPQGVRGEPGPPGPAGAAGPAGNPGADGQPGAKGANGAPGIAGAPGFPGARGPSGPQ


MOD flake               GPSGPPGPK                  GEPGPTGIQGPPGPAGEEGKR

P02453 Bos taurus       GPSGPPGPKGNSGEPGAPGSKGDTGAKGEPGPTGIQGPPGPAGEEGKRGARGEPGPAGLP 480
B9VR88 Equus asinus     GPSGPPGPKGNSGEPGAPGNKGDTGAKGEPGPTGIQGPPGPAGEEGKRGARGEPGPTGLP 480
                        *******************.************************************:***

#86   Bovid             GPSGPPGPKGNSGEPGAPGSKGDTGAKGEPGPTGIQGPPGPAGEEGKRGARGEPGPAGLP
#69   Equus quagga      GPSGPPGPK                  GEPGPTGIQGPPGPAGEEGK    GEPGPTGLP
#1120 Bovid             GPSGPPGPKGNSGEPGAPGSKGDTGAK                        GEPGPAGLP
#1117 Bovid             GPSGPPGPK            GDTGAKGEPGPTGIQGPPGPAGEEGKR   GEPGPAGLP
#91   Bovid             GPSGPPGPKGNSGEPGAPGSKGDTGAKGEPGPTGIQGPPGPAGEEGKR   GEPGPTGLP


MOD flake               GFPGADGVAGPK      GAPGPAGPKGSPGEAGRPGEAGLPGAKGLT

P02453 Bos taurus       GPPGERGGPGSRGFPGADGVAGPKGPAGERGAPGPAGPKGSPGEAGRPGEAGLPGAKGLT 540
B9VR88 Equus asinus     GPPGERGGPGARGFPGADGVAGPKGPAGERGAPGPAGPKGSPGEAGRPGEAGLPGAKGLT 540
                        **********:*************************************************

#86   Bovid             GPPGER      GFPGADGVAGPK      GAPGPAGPKGSPGEAGRPGEAGLPGAKGLT
#69   Equus quagga      GPPGER      GFPGADGVAGPK      GAPGPAGPKGSPGEAGRPGEAGLPGAKGLT
#1120 Bovid             GPPGER      GFPGADGVAGPK      GAPGPAGPKGSPGEAGRPGEAGLPGAKGLT
#1117 Bovid             GPPGER      GFPGADGVAGPK      GAPGPAGPKGSPGEAGRPGEAGLPGAKGLT
#91   Bovid             GPPGER      GFPGADGVAGPK      GAPGPAGPKGSPGEAGRPGEAGLPGAKGLT


MOD flake               GSPGSPGPDGK                      GQAGVMGFPGPK

P02453 Bos taurus       GSPGSPGPDGKTGPPGPAGQDGRPGPPGPPGARGQAGVMGFPGPKGAAGEPGKAGERGVP 600
B9VR88 Equus asinus     GSPGSPGPDGKTGPPGPAGQDGRPGPPGPPGARGQAGVMGFPGPKGAAGEPGKAGERGVP 600
                        ************************************************************


#86   Bovid             GSPGSPGPDGKTGPPGPAGQDGRPGPPGPPGARGQAGVMGFPGPKGAAGEPGK    GVP
#69   Equus quagga      GSPGSPGPDGK                      GQAGVMGFPGPKGAAGEPGK    GVP
#1120 Bovid             GSPGSPGPDGKTGPPGPAGQDGRPGPPGPPGARGQAGVMGFPGPK
#1117 Bovid             GSPGSPGPDGKTGPPGPAGQDGRPGPPGPPGARGQAGVMGFPGPK            GVP
#91   Bovid             GSPGSPGPDGKTGPPGPAGQDGRPGPPGPPGARGQAGVMGFPGPKGAAGEPGKAGERGVP


MOD flake

P02453 Bos taurus       GPPGAVGPAGKDGEAGAQGPPGPAGPAGERGEQGPAGSPGFQGLPGPAGPPGEAGKPGEQ 660
B9VR88 Equus asinus     GPPGAVGPAGKDGEAGAQGPPGPAGPAGERGEQGPAGSPGFQGLPGPAGPPGESGKPGEQ 660
                        *****************************************************:******

#86   Bovid             GPPGAVGPAGKDGEAGAQGPPGPAGPAGERGEQGPAGSPGFQGLPGPAGPPGEAGKPGEQ
#69   Equus quagga      GPPGAVGPAGKDGEAGAQGPPGPAGPAGER
#1120 Bovid             GPPGAVGPAGK
#1117 Bovid             GPPGAVGPAGKDGEAGAQGPPGPAGPAGERGEQGPAGSPGFQGLPGPAGPPGEAGKPGEQ
#91   Bovid             GPPGAVGPAGKDGEAGAQGPPGPAGPAGER
```

```
MOD flake                                       GVQGPPGPAGPR

P02453 Bos taurus     GVPGDLGAPGPSGARGERGFPGERGVQGPPGPAGPRGANGAPGNDGAKGDAGAPGAPGSQ 720
B9VR88 Equus asinus   GVPGDLGAPGPSGARGERGFPGERGVQGPPGPAGPRGSNGAPGNDGAKGDAGAPGAPGSQ 720
                      *************************************:**********************

#86   Bovid           GVPGDLGAPGPSGAR   GFPGERGVQGPPGPAGPRGSNGAPGNDGAKGDAGAPGAPGSQ
#69   Equus quagga                            GVQGPPGPAGPR            GDAGAPGAPGSQ
#1120 Bovid                             GFPGERGVQGPPGPAGPR            GDAGAPGAPGSQ
#1117 Bovid           GVPGDLGAPGPSGAR   GFPGERGVQGPPGPAGPR            GDAGAPGAPGSQ
#91   Bovid                             GFPGERGVQGPPGPAGPR


MOD flake                         GAAGLPGPK         GADGAPGKDGVRGLTGPIGPPGPAGAPGDK

P02453 Bos taurus     GAPGLQGMPGERGAAGLPGPKGDRGDAGPKGADGAPGKDGVRGLTGPIGPPGPAGAPGDK 780
B9VR88 Equus asinus   GAPGLQGMPGERGAAGLPGPKGDRGDAGPKGADGSPGKDGVRGLTGPIGPPGPAGAPGDK 780
                      **********************************:*************************

#86   Bovid           GAPGLQGMPGER                              GLTGPIGPPGPAGAPGDK
#69   Equus quagga    GAPGLQGMPGERGAAGLPGPK                     GLTGPIGPPGPAGAPGDK
#1120 Bovid           GAPGLQGMPGER                  GADGAPGKDGVR
#1117 Bovid           GAPGLQGMPGERGAAGLPGPKGDR                  GLTGPIGPPGPAGAPGDK
#91   Bovid                       GAAGLPGPK         GADGAPGKDGVRGLTGPIGPPGPAGAPGDK


MOD flake             GEAGPSGPAGPTGARGAPGDRGEPGPPGPAGFAGPPGADGQPGAK

P02453 Bos taurus     GEAGPSGPAGPTGARGAPGDRGEPGPPGPAGFAGPPGADGQPGAKGEPGDAGAKGDAGPP 840
B9VR88 Equus asinus   GETGPSGPAGPTGARGAPGDRGEPGPPGPAGFAGPPGADGQPGAKGEPGDAGAKGDAGPP 840
                      **:*********************************************************

#86   Bovid           GEAGPSGPAGPTGARGAPGDRGEPGPPGPAGFAGPPGADGQPGAKGEPGDAGAKGDAGPP
#69   Equus quagga    GETGPSGPAGPTGAR      GEPGPPGPAGFAGPPGADGQPGAK         GDAGPP
#1120 Bovid
#1117 Bovid           GEAGPSGPAGPTGARGAPGDRGEPGPPGPAGFAGPPGADGQPGAK         GDAGPP
#91   Bovid           GEAGPSGPAGPTGARGAPGDRGEPGPPGPAGFAGPPGADGQPGAKGEPGDAGAKGDAGPP


MOD flake

P02453 Bos taurus     GPAGPAGPPGPIGNVGAPGPKGARGSAGPPGATGFPGAAGRVGPPGPSGNAGPPGPPGPA 900
B9VR88 Equus asinus   GPAGPAGPPGPIGSVGAPGPKGARGSAGPPGATGFPGAAGRVGPPGPSGNAGPPGPPGPV 900
                      *************.*********************************************.

#86   Bovid           GPAGPAGPPGPIGNVGAPGPK   GSAGPPGATGFPGAAGRVGPPGPSGNAGPPGPPGPA
#69   Equus quagga    GPAGPAGPPGPIGSVGAPGPK   GSAGPPGATGFPGAAGRVGPPGPSGNAGPPGPPGPV
#1120 Bovid                                   GSAGPPGATGFPGAAGRVGPPGPSGNAGPPGPPGPA
#1117 Bovid           GPAGPAGPPGPIGNVGAPGPK   GSAGPPGATGFPGAAGRVGPPGPSGNAGPPGPPGPA
#91   Bovid           GPAGPAGPPGPIGNVGAPGPKGARGSAGPPGATGFPGAAGRVGPPGPSGNAGPPGPPGPA


MOD flake

P02453 Bos taurus     GKEGSKGPRGETGPAGRPGEVGPPGPPGPAGEKGAPGADGPAGAPGTPGPQGIAGQRGVV 960
B9VR88 Equus asinus   GKEGGKGPRGETGPAGRPGEAGPPGPPGPAGEKGSPGADGPAGAPGTPGPQGIAGQRGVV 960
                      ****.***************.*************:*************************

#86   Bovid           GK    GPRGETGPAGRPGEVGPPGPPGPAGEKGAPGADGPAGAPGTPGPQGIAGQRGVV
#69   Equus quagga    GK       GETGPAGRPGEAGPPGPPGPAGEKGSPGADGPAGAPGTPGPQGIAGQRGVV
#1120 Bovid           GK       GETGPAGRPGEVGPPGPPGPAGEK                        GVV
#1117 Bovid           GK    GPRGETGPAGRPGEVGPPGPPGPAGEKGAPGADGPAGAPGTPGPQGIAGQRGVV
#91   Bovid           GK       GETGPAGRPGEVGPPGPPGPAGEKGAPGADGPAGAPGTPGPQGIAGQRGVV


MOD flake                  GFPGLPGPSGEPGK          GPPGPMGPPGLAGPPGESGREGAPGAE
```

```
P02453 Bos taurus     GLPGQRGERGFPGLPGPSGEPGKQGPSGASGERGPPGPMGPPGLAGPPGESGREGAPGAE 1020
B9VR88 Equus asinus   GLPGQRGERGFPGLPGPSGEPGKQGPSGASGERGPPGPVGPPGLAGPPGESGREGSPGAE 1020
                      **************************************:****************:****

#86   Bovid           GLPGQRGERGFPGLPGPSGEPGKQGPSGASGERGPPGPMGPPGLAGPPGESGREGAPGAE
#69   Equus quagga    GLPGQRGERGFPGLPGPSGEPGKQGPSGASGERGPPGPVGPPGLAGPPGESGR
#1120 Bovid           GLPGQR   GFPGLPGPSGEPGK                              EGAPGAE
#1117 Bovid           GLPGQR   GFPGLPGPSGEPGKQGPSGASGERGPPGPMGPPGLAGPPGESGREGAPGAE
#91   Bovid           GLPGQRGERGFPGLPGPSGEPGK                              EGAPGAE

MOD flake             GSPGRDGSPGAKGDRGETGPAGPPGAPGAPGAPGPVGPAGKSGDRGETGPAGPAGPIGPV

P02453 Bos taurus     GSPGRDGSPGAKGDRGETGPAGPPGAPGAPGAPGPVGPAGKSGDRGETGPAGPAGPIGPV 1080
B9VR88 Equus asinus   GSPGRDGSPGPKGDRGETGPAGPPGAPGAPGAPGPVGPAGKSGDRGEAGPAGPAGPIGPV 1080
                      ********** ************************************:************

#86   Bovid           GSPGR       GDRGETGPAGPPGAPGAPGAPGPVGPAGKSGDRGETGPAGPAGPIGPV
#69   Equus quagga                   GETGPAGPPGAPGAPGAPGPVGPAGKSGDRGEAGPAGPAGPIGPV
#1120 Bovid           GSPGRDGSPGAK   GETGPAGPPGAPGAPGAPGPVGPAGK    GETGPAGPAGPVGPV
#1117 Bovid           GSPGR       GDRGETGPAGPPGAPGAPGAPGPVGPAGKSGDRGETGPAGPAGPIGPV
#91   Bovid           GSPGRDGSPGAKGDRGETGPAGPPGAPGAPGAPGPVGPAGKSGDRGETGPAGPAGPIGPV

MOD flake GAR                                       GFSGLQGPPGPPGSPGEQGPSGASGPAGPR

P02453 Bos taurus     GARGPAGPQGPRGDKGETGEQGDRGIKGHRGFSGLQGPPGPPGSPGEQGPSGASGPAGPR 1140
B9VR88 Equus asinus   GARGPAGPQGPRGDKGETGEQGDRGIKGHRGFSGLQGPPGPPGSPGEQGPSGASGPAGPR 1140
                      ************************************************************

#86   Bovid           GARGPAGPQGPR                  GFSGLQGPPGPPGSPGEQGPSGASGPAGPR
#69   Equus quagga    GARGPAGPQGPR                           GPPGSPGEQGPSGASGPAGPR
#1120 Bovid           GAR
#1117 Bovid           GARGPAGPQGPR                  GFSGLQGPPGPPGSPGEQGPSGASGPAGPR
#91   Bovid           GARGPAGPQGPRGDK

MOD flake

P02453 Bos taurus     GPPGSAGSPGKDGLNGLPGPIGPPGPRGRTGDAGPAGPPGPPGPPGPPGPPSGGYDLSFL 1200
B9VR88 Equus asinus   GPPGSAGAPGKDGLNGLPGPIGPPGPRGRTGDAGPVGPPGPPGPPGPPGPPSAGFDFSFL 1200
                      *******:***************************.****************.*:*:***

#86   Bovid           GPPGSAGSPGKDGLNGLPGPIGPPGPR
#69   Equus quagga
#1120 Bovid
#1117 Bovid           GPPGSAGSPGKDGLNGLPGPIGPPGPR
##91   Bovid                     DGLNGLPGPIGPPGPR

MOD flake

P02453 Bos taurus     PQPPQEKAHDGGRYYR 1260
B9VR88 Equus asinus   PQPPQEKSHDGGRYYR 1260
                      *******:********

#86   Bovid
#69   Equus quagga
#1120 Bovid
#1117 Bovid
##91   Bovid
```

Fig. H. Alignment of the matched peptides in the MOD flake to the Collagen alpha-2(I) chain from *Bos taurus* (P02465) and *Equus asinus* (B9VR89). Peptides identified in the MOD flake sample were manually aligned over the automatically generated alignment (CLUSTAL O(1.2.0) multiple sequence alignment, of the two collagen alpha-2(I) chain from *Bos taurus* (P02465) and *Equus asinus* (B9VR89). Peptides from MOD flake are reported in bold. An * (asterisk) indicates positions which have a single, fully conserved residue; : (colon) indicates conservation between groups of strongly similar properties; . (period) indicates conservation between groups of weakly similar properties. Under the alignment, peptides matched in samples from bones of some Bovids and *Equus quagga* remains recovered from Sibudu layer MOD, are reported. Underlined sequences are those identified in the error tolerant database search and the modified residue is indicated in red. Numbering refers to the precursor polypeptide chain while only the sequence of the mature protein is reported. X stands for either Ile or Leu that cannot be distinghished as substitution since are isobaric.

```
MOD flake                                                          GPPGASGAPGPQGFXGPPGEPGE

P02465 Bos taurus                        QFDAKGGGPGPMGLMGPRGPPGASGAPGPQGFQGPPGEPGE 120
B9VR89 Equus asinus                      QFDAKGGGPGPMGLMGPRGPPGASGAPGPQGFQGPAGEPGE 120
                                         *********************************** *****

#86   Bovid                                                        GPPGASGAPGPQGFQGPPGEPGE
#69   Equus quagga
#1120 Bovid
#1117 Bovid                                                        GPPGASGAPGPQGFQGPPGEPGE
#91   Bovid

MOD flake             PGQTGPAGAR                        GVVGPQGAR

P02465 Bos taurus     PGQTGPAGARGPPGPPGKAGEDGHPGKPGRPGERGVVGPQGARGFPGTPGLPGFKGIRGH 180
B9VR89 Equus asinus   PGQTGPAGARGPPGPPGKAGEDGHPGKPGRPGERGVVGPQGARGFPGTPGLPGFKGIRGH 180
                      ************************************************************

#86   Bovid           PGQTGPAGARGPPGPPGK                GVVGPQGARGFPGTPGLPGFK   GH
#69   Equus quagga              GPPGPPGK                GVVGPQGARGFPGTPGLPGFK
#1120 Bovid                     GPPGPPGK                GVVGPQGAR
#1117 Bovid           PGQTGPAGARGPPGPPGKAGEDGHPGKPGRPGERGVVGPQGARGFPGTPGLPGFK
#91   Bovid                     GPPGPPGK                GVVGPQGAR

MOD flake                                                       VGAPGPAGAR

P02465 Bos taurus     NGLDGLKGQPGAPGVKGEPGAPGENGTPGQTGARGLPGERGRVGAPGPAGARGSDGSVGP 240
B9VR89 Equus asinus   KGLDGLKGQPGAPGVKGEPGAPGENGTPGQAGARGLPGERGRVGAPGPAGARGSDGSVGP 240
                      :*****************************:*****************************

#86   Bovid           NGLDGLKGQPGAPGVKGEPGAPGENGTPGQTGARGLPGER  VGAPGPAGARGSDGSVGP
#69   Equus quagga           GQPGAPGVK                  GLPGER  VGAPGPAGARGSDGSVGP
#1120 Bovid                                             GLPGER  VGAPGPAGAR
#1117 Bovid                  GQPGAPGVKGEPGAPGENGTPGQTGARGLPGER  VGAPGPAGARGSDGSVGP
#91   Bovid                                                     VGAPGPAGAR

MOD flake

P02465 Bos taurus     VGPAGPIGSAGPPGFPGAPGPKGELGPVGNPGPAGPAGPRGEVGLPGLSGPVGPPGNPGA 300
B9VR89 Equus asinus   VGPAGPIGSAGPPGFPGAPGPKGELGPVGNPGPAGPAGPRGEVGLPGLSGPVGPPGNPGA 300
                      ************************************************************

#86   Bovid           VGPAGPIGSAGPPGFPGAPGPKGELGPVGNPGPAGPAGPRGEVGLPGLSGPVGPPGNPGA
#69   Equus quagga    VGPAGPIGSAGPPGFPGAPGPKGELGPVGNPGPAGPAGPRGEVGLPGLSGPVGPPGNPGA
#1120 Bovid
#1117 Bovid           VGPAGPIGSAGPPGFPGAPGPKGELGPVGNPGPAGPAGPRGEVGLPGLSGPVGPPGNPGA
#91   Bovid                                   GELGPVGNPGPAGPAGPR
```

```
MOD flake                    GAAGLPGVAGAPGLPGPR

P02465 Bos taurus     NGLPGAKGAAGLPGVAGAPGLPGPRGIPGPVGAAGATGARGLVGEPGPAGSKGESGNKGE 360
B9VR89 Equus asinus   NGLTGAKGAAGLPGVAGAPGLPGPRGIPGPAGAAGATGARGLVGEPGPAGSKGESGNKGE 360
                      *** **************************.*****************************

#86   Bovid           NGLPGAKGAAGLPGVAGAPGLPGPRGIPGPVGAAGATGARGLVGEPGPAGSKGESGNKGE
#69   Equus quagga    NGLTGAKGAAGLPGVAGAPGLPGPRGIPGPAGAAGATGARGLVGEPGPAGSK
#1120 Bovid                                    GIPGPVGAAGATGAR
#1117 Bovid           NGLPGAKGAAGLPGVAGAPGLPGPRGIPGPVGAAGATGARGLVGEPGPAGSKGESGNKGE
#91   Bovid                  GAAGLPGVAGAPGLPGPRGIPGPVGAAGATGARGLVGEPGPAGSK

MOD flake                                GSTGEIGPAGPPGPPGLR      GLPGADGR

P02465 Bos taurus     PGAVGQPGPPGPSGEEGKRGSTGEIGPAGPPGPPGLRGNPGSRGLPGADGRAGVMGPAGS 420
B9VR89 Equus asinus   PGAAGPQGPPGPSGEEGKRGPNGEPGSTGPAGPPGLRGSPGSRGLPGADGRAGVMGPAGS 420
                      ***.*  ************* .** * :** *******.*********************

#86   Bovid           PGAVGQPGPPGPSGEEGKRGSTGEIGPAGPPGPPGLR      GLPGADGRA
#69   Equus quagga                      RGPNGEPGSTGPAGPPGLR              AGVMGPAGS
#1120 Bovid                                                      GLPGADGRAGVMGPAGS
#1117 Bovid           PGAVGQPGPPGPSGEEGKRGSTGEIGPAGPPGPPGLR      GLPGADGRAGVMGPAGS
#91   Bovid                              GSTGEIGPAGPPGPPGLR      GLPGADGRAGVMGPAGS

MOD flake              GATGPAGVR                                EGPVGLPGIDGRPGPIGP

P02465 Bos taurus     RGATGPAGVRGPNGDSGRPGEPGLMGPRGFPGSPGNIGPAGKEGPVGLPGIDGRPGPIGP 480
B9VR89 Equus asinus   RGATGPAGVRGPNGDSGRPGEPGLMGPRGFPGSPGNIGPAGKEGPVGLPGIDGRPGPIGP 480
                      ************************************************************

#86   Bovid                     GPNGDSGRPGEPGLMGPRGFPGSPGNIGPAGKEGPVGLPGIDGRPGPIGP
#69   Equus quagga    RGATGPAGVRGPNGDSGRPGEPGLMGPRGFPGSPGNIGPAGKEGPVGLPGIDGRPGPIGP
#1120 Bovid           RGATGPAGVR                  GFPGSPGNIGPAGK
#1117 Bovid           RGATGPAGVR                  GFPGSPGNIGPAGKEGPVGLPGIDGRPGPIGP
#91   Bovid           R                           GFPGSPGNIGPAGK

MOD flake             AGARGEPGNIGFPGPK

P02465 Bos taurus     AGARGEPGNIGFPGPKGPSGDPGKAGEKGHAGLAGARGAPGPDGNNGAQGPPGLQGVQGG 540
B9VR89 Equus asinus   AGARGEPGNIGFPGPKGPTGEPGKPGDKGHAGLAGARGAPGPDGNNGAQGPPGPQGVQGG 540
                      ******************:*:*** *:************************** ******

#86   Bovid           AGARGEPGNIGFPGPK            GHAGLAGARGAPGPDGNNGAQGPPGLQGVQGG
#69   Equus quagga    AGARGEPGNIGFPGPKGPTGEPGKPGDK         GAPGPDGNNGAQGPPGPQGVQGG
#1120 Bovid               GEPGNIGFPGPK
#1117 Bovid           AGARGEPGNIGFPGPK            GHAGLAGARGAPGPDGNNGAQGPPGLQGVQGG
#91   Bovid               GEPGNIGFPGPK

MOD flake                                            GIPGEFGLPGPAGAR   GPPGESGAAGP

P02465 Bos taurus     KGEQGPAGPPGFQGLPGPAGTAGEAGKPGERGIPGEFGLPGPAGARGERGPPGESGAAGP 600
B9VR89 Equus asinus   KGEQGPAGPPGFQGLPGPAGTAGEVGKPGERGLPGEFGLPGPAGARGERGPPGESGAAGP 600
                      ************************.*******:***************************

#86   Bovid           KGEQGPAGPPGFQGLPGPAGTAGEAGKPGERGIPGEFGLPGPAGARGERGPPGESGAAGP
#69   Equus quagga    KGEQGPAGPPGFQGLPGPAGTAGEVGKPGERGLPGEFGLPGPAGAR   GPPGESGAAGP
#1120 Bovid                                                            GPPGESGAAGP
#1117 Bovid           KGEQGPAGPPGFQGLPGPAGTAGEAGKPGERGIPGEFGLPGPAGVRGERGPPGESGAAGP
#91   Bovid                                                         GERGPPGESGAAGP

MOD flake             TGPIGSR
```

```
P02465 Bos taurus     TGPIGSRGPSGPPGPDGNKGEPGVVGAPGTAGPSGPSGLPGERGAAGIPGGKGEKGETGL 660
B9VR89 Equus asinus   AGPIGSRGPSGPPGPDGNKGEPGVLGAPGTAGPSGPSGLPGERGAAGIPGGKGEKGETGL 660
                      :***********************:***********************************

#86   Bovid           TGPIGSRGPSGPPGPDGNKGEPGVVGAPGTAGPSGPSGLPGERGAAGIPGGK   GETGL
#69   Equus quagga    AGPIGSR                                    GAAGIPGGK
#1120 Bovid           TGPIGSR                                    GAAGIPGGK
#1117 Bovid           TGPIGSRGPSGPPGPDGNKGEPGVVGAPGTAGPSGPSGLPGERGAAGIPGGKGEKGETGL
#91   Bovid           TGPIGSR                                    GAAGIPGGKGEK


MOD flake

P02465 Bos taurus     RGDIGSPGRDGARGAPGAIGAPGPAGANGDRGEAGPAGPAGPAGPRGSPGERGEVGPAGP 720
B9VR89 Equus asinus   RGEIGNPGRDGARGAPGAVGAPGPAGANGDRGEAGAAGPAGPAGPRGSPGERGEVGPAGP 720
                      **:**.************:**************** ************************

#86   Bovid           R            GAPGAVGAPGPAGANGDRGEAGPAGXAGPAGPR      GEVGPAGP
#69   Equus quagga     GEIGNPGRDGARGAPGAVGAPGPAGANGDRGEAGAAGPAGPAGPR      GEVGPAGP
#1120 Bovid
#1117 Bovid           R            GAPGAVGAPGPAGANGDRGEAGPAGPAGPAGPR      GEVGPAGP
#91   Bovid                        GATGAIGAPGPAGANGDRGEAGPAGPAGPAGPR      GEVGPAGP

MOD flake                                                                  GDGGP

P02465 Bos taurus     NGFAGPAGAAGQPGAKGERGTKGPKGENGPVGPTGPVGAAGPSGPNGPPGPAGSRGDGGP 780
B9VR89 Equus asinus   NGFAGPAGAAGQPGAKGERGTKGPKGENGPVGPTGPVGAAGPSGPNGPPGPAGSRGDGGP 780
                      ************************************************************

#86   Bovid           NGFAGPAGAAGQPGAKGER   GPKGENGPVGPTGPVGAAGPSGPNGPPGPAGSRGDGGP
#69   Equus quagga    NGFAGPAGAAGQPGAKGER      GENGPVGPTGPVGAAGPSGPNGPPGPAGSRGDGGP
#1120 Bovid                                                                  GDGGP
#1117 Bovid           NGFAGPAGAAGQPGAKGER   GPKGENGPVGPTGPVGAAGPSGPNGPPGPAGSRGDGGP
#91   Bovid           NGFAGPAGAAGQPGAKGER      GENGPVGPTGPVGAAGPSGPNGPPGPAGSRGDGGP


MOD flake             PGATGFPGAAGR

P02465 Bos taurus     PGATGFPGAAGRTGPPGPSGISGPPGPPGPAGKEGLRGPRGDQGPVGRSGETGASGPPGF 840
B9VR89 Equus asinus   PGVTGFPGAAGRTGPPGPSGISGPPGPPGAAGKEGLRGPRGDQGPVGRAGETGASGPPGF 840
                      **.************************** *******************:**********

#86   Bovid           PGATGFPGAAGRTGPPGPSGISGPPGPPGPAGKEGLRGPRGDQGPVGR
#69   Equus quagga    PGVTGFPGAAGRTGPPGPSGISGPPGPPGAAGK       GDQGPVGRAGETGASGPPGF
#1120 Bovid           PGATGFPGAAGR                            GDQGPVGR
#1117 Bovid           PGATGFPGAAGRTGPPGPSGISGPPGPPGPAGK       GDQGPVGR
#91   Bovid           PGATGFPGAAGRTGPPGPSGISGPPGPPGPAGK    GPRGDQGPVGR


MOD flake                                                     GLPGVAGSVGEPGPLGIAGP

P02465 Bos taurus     VGEKGPSGEPGTAGPPGTPGPQGLLGAPGFLGLPGSRGERGLPGVAGSVGEPGPLGIAGP 900
B9VR89 Equus asinus   AGEKGPSGEPGTAGPPGTPGPQGLLGAPGILGLPGSRGERGLPGVAGSLGEPGPLGIAGP 900
.                     ****************************:******************:***********

#86   Bovid               GPSGEPGTAGPPGTPGPQGLLGXPGFLGLPGSR GLPGVAGSVGEPGPLGIAGP
#69   Equus quagga    AGEK                                    GLPGVAGSLGEPGPLGIAGP
#1120 Bovid
#1117 Bovid               GPSGEPGTAGPPGTPGPQGLLGXPGFLGLPGSR   GLPGVAGSVGEPGPLGIAGP
#91   Bovid


MOD flake             PGAR

P02465 Bos taurus     PGARGPPGNVGNPGVNGAPGEAGRDGNPGNDGPPGRDGQPGHKGERGYPGNAGPVGAAGA 960
B9VR89 Equus asinus   PGARGPPGAVGAPGVNGAPGEAGRDGNPGSDGPPGRDGQPGHKGERGYPGNAGPVGAVGA 960
```

```
                      ******** ** *****************.***************************.**

#86   Bovid           PGARGPPGNVGNPGVNGAPGEAGRDGNPGNDGPPGRDGQPGHK   GYPGNAGPVGTAGA
#69   Equus quagga    PGARGPPGAVGAPGVNGAPGEAGR
#1120 Bovid
#1117 Bovid           PGARGPPGNVGNPGVNGAPGEAGRDGNPGNDGPPGR          GYPGNAGPVGTTGA
#91   Bovid


MOD flake

P02465 Bos taurus     PGPQGPVGPVGKHGNRGEPGPAGAVGPAGAVGPRGPSGPQGIRGDKGEPGDKGPRGLPGL 1020
B9VR89 Equus asinus   PGPHGPVGPTGKHGNRGEPGPVGSVGPVGAVGPRGPSGPQGVRGDKGEPGDKGPRGLPGI 1020
                      ***:*****.***********.*:***.*************:*****************:

#86   Bovid           PGPQGPVGPVGKHGNRGEPGPAGAVGPAGAVGPRGPSGPQGIRGDK         GLPGL
#69   Equus quagga                    GEPGPVGSVGPVGAVGPRGPSGPQGVR
#1120 Bovid                           GEPGPAGAVGPAGAVGPRGPSGPQGIR
#1117 Bovid           PGPQGPVGPVGK    GEPGPAGAVGPAGAVGPRGPSGPQGIR            GLPGL
#91   Bovid                           GEPGPAGAVGPAGAVGPRGPSGPQGIR


MOD flake                                            GPAGPSGPAGKDGR

P02465 Bos taurus     KGHNGLQGLPGLAGHHGDQGAPGAVGPAGPRGPAGPSGPAGKDGRIGQPGAVGPAGIRGS 1080
B9VR89 Equus asinus   KGHNGLQGLPGLAGQHGDQGAPGSVGPAGPRGPAGPTGPVGKDGRSGQPGTVGPAGVRGS 1080
                      **************:********:************:**.***** ****:*****:***

#86   Bovid           KGHNGLQGLPGLAGHHGDQGAPGAVGPAGPRGPAGPSGPAGK   IGQPGAVGPAGIR
#69   Equus quagga                                   GPAGPTGPVGK   SGQPGTVGPAGVR
#1120 Bovid                                          GPAGPSGPAGKDGRIGQPGAVGPAGIR
#1117 Bovid           K                              GPAGPSGPAGKDGRIGQPGAVGPAGIR
#91   Bovid                                          GPAGPSGPAGK   IGQPGAVGPAGIR


MOD flake

P02465 Bos taurus     QGSQGPAGPPGPPGPPGPPGP 1140
B9VR89 Equus asinus   QGSQGPAGPPGPPGPPGPPGP 1140
                      *********************

#86   Bovid
#69   Equus quagga
#1120 Bovid
#1117 Bovid
#91   Bovid
```

References

1. Andreotti A, Bonaduce I, Colombini MP, Gautier G, Modugno F, Ribechini E. Combined GC/MS analytical procedure for the characterization of glycerolipid, waxy, resinous, and proteinaceous materials in a unique paint microsample. Anal Chem. 2006; 78: 4490-4500.
2. Lluveras A, Bonaduce I, Andreotti A, Colombini MP. GC/MS analytical procedure for the characterization of glycerolipids, natural waxes, terpenoid resins, proteinaceous and polysaccharide materials in the same paint microsample avoiding interferences from inorganic media. Anal Chem. 2010; 82: 376–386.
3. Villa P**,** Soriano S,Tsanova T, Degano I, Higham TFG, d'Errico F, Backwell L, Lucejko JJ, Colombini MP, Beaumont PB. Border Cave and the beginning of the Later Stone Age in South Africa. Proc Natl Acad Sci USA. 2012**;** 109 (33): 13208-13213.
4. Jacobs Z, Roberts RG, Galbraith RF, Deacon HJ, Grün R, Mackay A, et al. Ages for the Middle Stone Age of Southern Africa: Implications for Human Behavior and Dispersal. Science. 2008; 322, 733-735.
5. Gibson NE, Wadley L, Williamson BS. Microscopic residues as evidence of hafting on backed tools from 60,000 to 68,000 year-old Howiesons Poort layers of Rose Cottage Cave, South Africa. South Afr Humanit. 2004; 16:1-11.
6. Jacobs Z, Wintle AG, Duller GAT, Roberts RG, Wadley L.New ages for the post-Howiesons Poort, late and final Middle Stone Age at Sibudu, South Africa. J Archaeol Sci. 2008; 35: 1790-1807.
7. Allott LF. Archaeological charcoal as a window on paleovegetation and wood-use during the Middle Stone Age at Sibudu Cave. South Afr Humanit. 2006; 18: 173-201.
8. Leo G, Cartechini L, Pucci P, Sgamellotti A, Marino G, Birolo L . Proteomic strategies for the identification of proteinaceous binders in paintings. Anal Bioanal Chem. 2009; 395: 2269-2280.
9. Brain CK. The hunters or the hunted? An introduction to African cave taphonomy. Chicago: University of Chicago Press; 1981.
